# Structure-based protein function prediction using graph convolutional networks

**DOI:** 10.1038/s41467-021-23303-9

**Published:** 2021-05-26

**Authors:** Vladimir Gligorijević, P. Douglas Renfrew, Tomasz Kosciolek, Julia Koehler Leman, Daniel Berenberg, Tommi Vatanen, Chris Chandler, Bryn C. Taylor, Ian M. Fisk, Hera Vlamakis, Ramnik J. Xavier, Rob Knight, Kyunghyun Cho, Richard Bonneau

**Affiliations:** 1 Center for Computational Biology, Flatiron Institute, New York, NY USA; 2 Department of Pediatrics, University of California San Diego, La Jolla, CA USA; 3 Malopolska Centre of Biotechnology, Jagiellonian University, Krakow, Poland; 4 Courant Institute of Mathematical Sciences, Department of Computer Science, New York University, New York, NY USA; 5 Broad Institute of MIT and Harvard, Cambridge, MA USA; 6 The Liggins Institute, University of Auckland, Auckland, New Zealand; 7 Biomedical Sciences Graduate Program, University of California San Diego, La Jolla, CA USA; 8 Scientific Computing Core, Flatiron Institute, Simons Foundation, New York, NY USA; 9 Center for Computational and Integrative Biology, Massachusetts General Hospital and Harvard Medical School, Boston, MA USA; 10 Gastrointestinal Unit, and Center for the Study of Inflammatory Bowel Disease, Massachusetts General Hospital and Harvard Medical School, Boston, MA USA; 11 Center for Microbiome Informatics and Therapeutics, MIT, Cambridge, MA USA; 12 Center for Microbiome Innovation, University of California San Diego, La Jolla, CA USA; 13 Department of Computer Science and Engineering, University of California San Diego, La Jolla, CA USA; 14 Center for Data Science, New York University, New York, NY USA; 15 CIFAR Azrieli Global Scholar, New York, NY USA; 16 Center for Genomics and Systems Biology, Department of Biology, New York University, New York, NY USA

**Keywords:** Machine learning, Protein function predictions, Protein structure predictions

## Abstract

The rapid increase in the number of proteins in sequence databases and the diversity of their functions challenge computational approaches for automated function prediction. Here, we introduce DeepFRI, a Graph Convolutional Network for predicting protein functions by leveraging sequence features extracted from a protein language model and protein structures. It outperforms current leading methods and sequence-based Convolutional Neural Networks and scales to the size of current sequence repositories. Augmenting the training set of experimental structures with homology models allows us to significantly expand the number of predictable functions. DeepFRI has significant de-noising capability, with only a minor drop in performance when experimental structures are replaced by protein models. Class activation mapping allows function predictions at an unprecedented resolution, allowing site-specific annotations at the residue-level in an automated manner. We show the utility and high performance of our method by annotating structures from the PDB and SWISS-MODEL, making several new confident function predictions. DeepFRI is available as a webserver at https://beta.deepfri.flatironinstitute.org/.

## Abstract (web summary)

The rapid increase in the number of proteins in sequence databases and the diversity of their functions challenge computational approaches for automated function prediction. Here, the authors introduce DeepFRI, a Graph Convolutional Network for predicting protein functions by leveraging sequence features extracted from a protein language model and protein structures.

## Introduction

Proteins fold into 3-dimensional structures to carry out a wide variety of functions within the cell^1^. Even though many functional regions of proteins are disordered, the majority of domains fold into specific and ordered three-dimensional conformations^2–6^. In turn, the structural features of proteins determine a wide range of functions: from binding specificity and conferring mechanical stability, to catalysis of biochemical reactions, transport, and signal transduction. There are several widely used classification schemes that organize these myriad protein functions including the Gene Ontology (GO) Consortium^7^, Enzyme Commission (EC) numbers^8^, Kyoto Encyclopedia of Genes and Genomes (KEGG)^9^, and others. For example, GO classifies proteins into hierarchically related functional classes organized into three different ontologies: Molecular Function (MF), Biological Process (BP), and Cellular Component (CC), to describe different aspects of protein functions.

The advent of efficient low-cost sequencing technologies and advances in computational methods (e.g., gene prediction) have resulted in a massive growth in the number of sequences available in key protein sequence databases like the UniProt Knowledgebase (UniProtKB)^10^. UniProt currently contains over 100 million sequences, only about 0.5% of which are manually annotated (UniProtKB/Swiss-Prot). Due to considerations of scale, design, and costs of experiments to verify a function, it is safe to posit that most proteins with unknown function (i.e., hypothetical proteins) are unlikely to be experimentally characterized. Understanding the functional roles and studying the mechanisms of newly discovered proteins is one of the most important biological problems in the post-genomic era. In parallel to the growth of sequence data, advances in experimental and computational techniques in structural biology has made the three-dimensional structures of many proteins available^11–18^. The Protein Data Bank (PDB)^19^, a repository of three-dimensional structures of proteins, nucleic acids, and complex assemblies, has experienced significant recent growth, reaching almost 170,000 entries. Large databases of comparative models such as SWISS-MODEL also provide valuable resources for studying structure–function relationships^13,20^.

To address the sequence-function gap many computational methods have been developed with the goal to automatically predict protein function. Further, related work is directed at predicting function in a site- or domain-specific manner^21–24^. Traditional machine learning classifiers, such as support vector machines, random forests, and logistic regression have been used extensively for protein function prediction. They have established that integrative prediction schemes outperform homology-based function transfer^25,26^ and that integration of multiple gene- and protein-network features typically outperform sequence-based features even though network features are often incomplete or unavailable. Systematic blind prediction challenges, such as the Critical Assessment of Functional Annotation (CAFA1^27^, CAFA2^28^, and CAFA3^29^) and MouseFunc^30^, are critical in the development of these methods and have shown that integrative machine learning and statistical methods outperform traditional sequence alignment-based methods (e.g., BLAST)^26^. However, the top-performing CAFA methods typically rely strongly on manually-engineered features constructed from either text, sequence, biological networks, or protein structure^31^. In most cases, for newly sequenced proteins, or proteins of poorly studied organisms these features are difficult to obtain because of limited information (e.g., no text features or biological network available). Here, we focus on methods that take sequence and sequence-based features (such as predicted structure) as inputs and do not focus on, or compare to, the many methods that rely on protein networks like GeneMANIA^32^, Mashup^33^, DeepNF^34^, and other integrative network prediction methods. As a result, we present a method applicable to hundreds of thousands of sequences of proteins from unknown organisms, lacking the required network data.

In the last decade, deep learning has led to unprecedented improvements in performance of methods tackling a broad spectrum of problems, ranging from learning protein sequence embeddings for contact map prediction^35^ to predicting protein structure^36,37^ and function^38^. In particular, convolutional neural networks (CNN)^39^, the state-of-the-art in computer vision, have shown tremendous success in addressing problems in computational biology. They have enabled task-specific feature extraction directly from protein sequence (or the corresponding 3D structure), overcoming the limitations of standard feature-based machine learning (ML) methods. The majority of sequence-based protein function prediction methods use 1D CNNs, or variations thereof, that search for recurring spatial patterns within a given sequence and converts them hierarchically into complex features using multiple convolutional layers. Recent work has employed 3D CNNs to extract features from protein structural data^40,41^. Although these works demonstrate the utility of structural features, storing and processing explicit 3D representations of protein structure at high resolution is not memory efficient, since most of the 3D space is unoccupied by protein structure. In contrast, geometric deep learning methods^42,43^, and more specifically graph convolutional networks (GCNs)^44^, overcome these limitations by generalizing convolutional operations on more efficient graph-like molecular representations. GCNs have shown tremendous success in various problems ranging from learning features for quantitative structure-activity relationship (QSAR) models^45^, to predicting biochemical activity of drugs^46^, to predicting interfaces between pairs of proteins^47^.

**Fig. 1:**
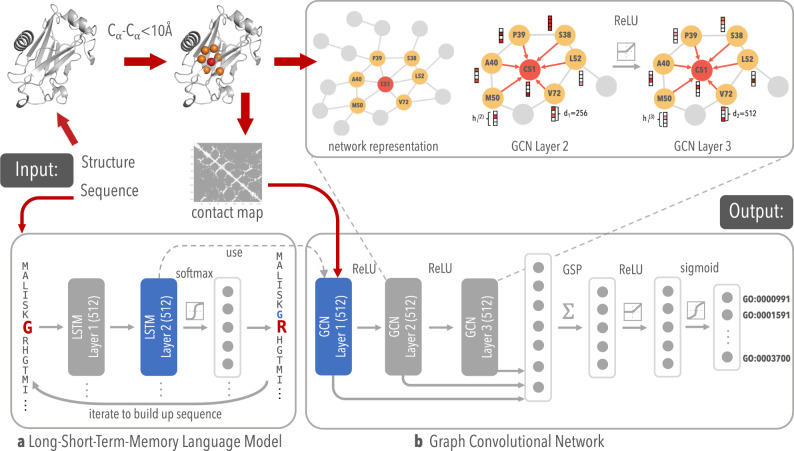
Schematic method overview. **a** LSTM language model, pre-trained on ~10 million Pfam protein sequences, used for extracting residue-level features of PDB sequence. **b** Our GCN with three graph convolutional layers for learning complex structure–function relationships.

Here, we describe a method based on GCNs for functionally annotating proteins and detecting functional regions in proteins, termed Deep Functional Residue Identification (DeepFRI), that outperforms current methods and scales to the size of current repositories of sequence information. Our model has a two-stage architecture that takes as input a protein structure and a sequence representation from a pre-trained, task-agnostic language model, represented as graphs derived from amino acid interactions in the 3D structure. The model outputs probabilities for each function (see Fig. 1) and identifies residues important for function prediction by using the gradient-weighted Class Activation Map (grad-CAM)^48^ approach, that we adapted for post-training analysis of GCNs. We provide several examples where we automatically and correctly identify functional sites for various functions where binding and catalytic sites are known.

## Results

### DeepFRI combines protein structure and pre-trained sequence embeddings in a GCN

In the past few years, it has been shown that features extracted from pre-trained, task-agnostic, language models can significantly increase classification performance in many natural language processing^49^ and biological problems^35^. Here, we use a similar approach for extracting features from sequences and learning protein representations. The first stage of our method is a self-supervised language model with a recurrent neural network architecture with long short-term memory (LSTM-LM)^50^. The language model is pre-trained on a set of protein domain sequences from the protein families database (Pfam)^51^, and is used for extracting residue-level features from PDB sequences (see Fig. 1a). The second stage is a GCN that uses a deep architecture to propagate the residue-level features between residues that are proximal in the structure and construct final protein-level feature representations (see Fig. 1b).

We train the LSTM-LM on a corpus of around 10 million protein domain sequences from Pfam^51^. Our LSTM-LM is trained to predict an amino acid residue in the context of its position in a protein sequence (see the “Methods” section for details). During the training of the GCN the parameters of the LSTM-LM are fixed; i.e., the LSTM-LM stage is only used as a sequence feature extractor. The residue-level features constructed for sequences, together with contact maps, are used as an input for the second stage of our method. Each layer of the graph convolution stage takes both an adjacency matrix and the residue-level features described above, and outputs the residue-level features in the next layer. We explore different types of graph convolutions, including the most widely used Kipf & Welling graph convolutional layer (GraphConv)^44^, Chebyshev spectral graph convolutions (ChebConv)^52^, SAmple and aggreaGatE convolutions (SAGEConv)^53^, Graph Attention (GAT)^54^, and a combination of different graph convolutional layers with different propagation rules (MultiGraphConv)^55^. Our comparison between different graph convolution formulations is shown in the “Methods” section and Supplementary Fig. 1. Three layers of MultiGraphConv or GAT often result in the best performance across many of our experiments. The GCN protein representation is obtained by concatenating features from all layers of this GCN into a single feature matrix and is subsequently fed into two fully connected layers to produce the final protein function predictions for all terms (see “Methods” for details on GCN architecture).

We train different models to predict GO terms (one model for each branch of the GO: molecular function, cellular component, biological process) and EC numbers. The GO terms are selected to have at least 50 and not more than 5000 training examples, whereas EC numbers are selected from levels 3 and 4 of the EC tree as they are the most specific descriptors of the enzymatic functions. We evaluate the function prediction performance by two measures commonly used in the CAFA challenges^27^ (see “Methods”): (1) protein-centric maximum *F*-score (*F*_max_) which measures the accuracy of assigning GO terms/EC numbers to a protein, and is computed as a harmonic mean of the precision and recall; and (2) term-centric area under precision-recall (AUPR) curve, which measures the accuracy of assigning proteins to different GO terms/EC numbers. When reporting the overall performance of a method the AUPR and *F*_max_ scores are averaged over all GO terms and all proteins in the test set, respectively. To compare different methods we also report the precision-recall curves representing the average precision and recall at the different values of the decision threshold *t* ∈ [0, 1].

This architecture leads to the main advantage of our method, that it convolves features over residues that are distant in the primary sequence, but close to each other in the 3D space, without having to learn these functionally relevant proximities from the data. Such an operation, implemented here using graph convolution, leads to better protein feature representations and ultimately to more accurate function predictions as shown in Supplementary Fig. 2. These results illustrate the importance of both graph convolutions and protein language model features as components of DeepFRI. Specifically, DeepFRI outperforms a baseline model which only takes into account contact maps in combination with simple one-hot sequence encoding, indicating that the LSTM-LM features significantly boost the predictive power compared to simplified residue feature representation. Moreover, by comparing DeepFRI with a baseline model that takes only language model features into account, we show the importance of protein structures and the effect of the long-range connections in the predictive performance of DeepFRI.

### DeepFRI improves performance when protein models are included in the training

We investigate the performance of DeepFRI trained only on experimentally determined, high-quality structures from the PDB. Further, to explore the possibility of including a large number of available protein models into the training, we examine the performance when homology models from SWISS-MODEL are included in the training procedure. This significantly increases the number of training samples per function and reduces the imbalance between positive and negative examples. GO term and EC number annotations for PDB and SWISS-MODEL chains are retrieved from SIFTS^56^ and UniProtKB/Swiss-Prot repositories, respectively. We report all our results on a test set consisting of only experimental PDB structures with varying degrees of sequence identity to the training set. For each annotated chain in PDB and SWISS-MODEL, we extract its sequence and construct its *C*_*α*_–*C*_*α*_ contact map (see “Methods” for data collection and pre-processing). We systematically explore the effect of different *C*_*α*_–*C*_*α*_ distance thresholds and different types of contact maps on the predictive power of DeepFRI (see Supplementary Fig. 3). We further explore different structure prediction methods for both training and prediction of newly observed sequences and find that using models from SWISS-MODEL during training greatly improves model comprehension and accuracy.

**Fig. 2:**
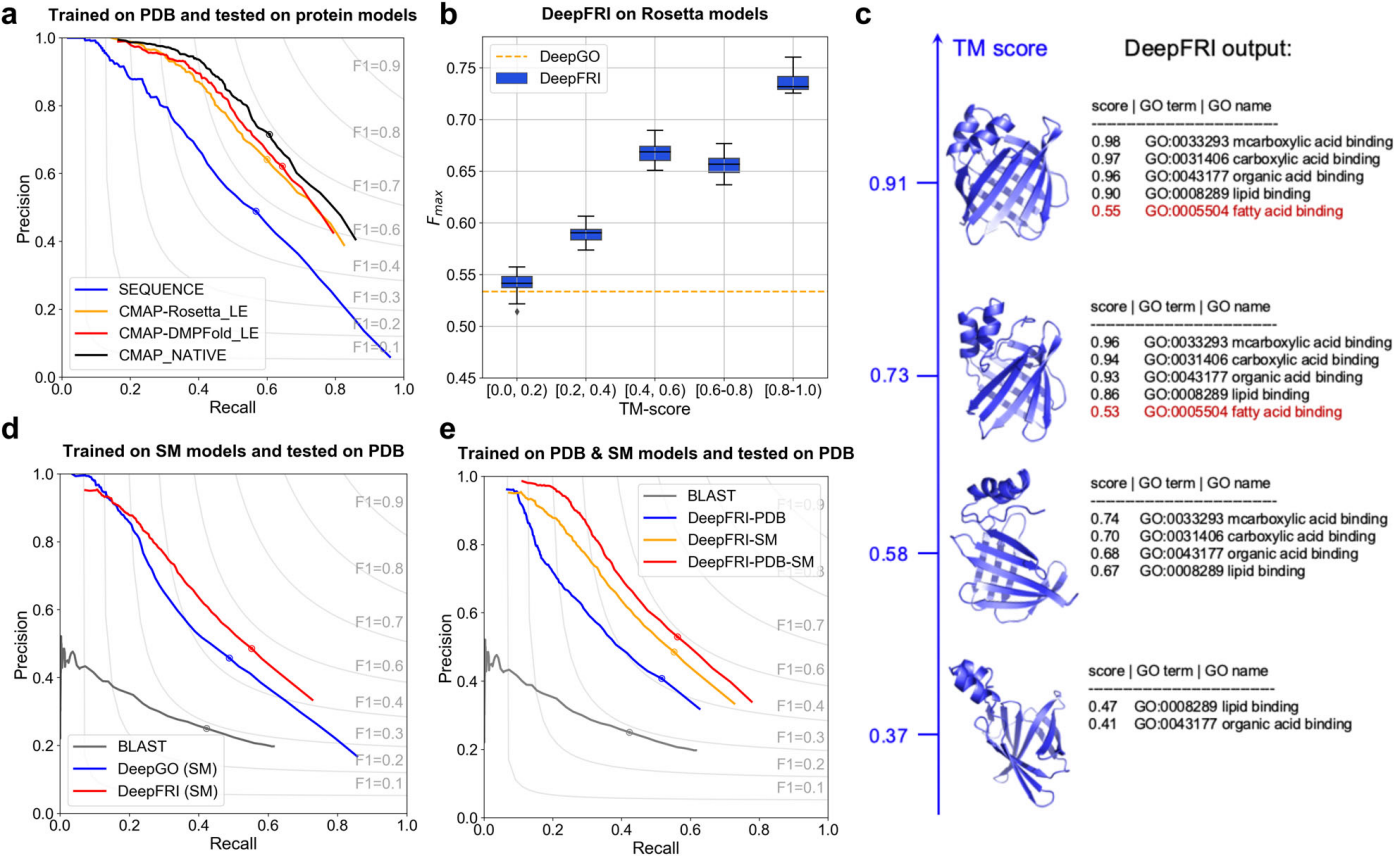
Performance of DeepFRI in predicting MF-GO terms of experimental structures and protein models. **a** Precision-recall curves showing the performance of DeepFRI on ~700 protein contact maps (PDB700 dataset) from NATIVE PDB structures (CMAP_NATIVE, black), their corresponding Rosetta-predicted lowest energy (LE) models (CMAP-Rosetta_LE, orange) and DMPfold lowest energy (LE) models (CMAP-DMPFold_LE, red), in comparison to the sequence-only CNN-based method (SEQUENCE, blue). All DeepFRI models are trained only on experimental PDB structures. **b** Distribution of protein-centric *F*_max_ score over 1500 different Rosetta models from the PDB700 dataset grouped by their TM-score computed against the native structures. Data are represented as boxplots with the center line representing the median, upper and lower edges of the boxes representing the interquartile range, and whiskers representing the data range (0.5 × interquartile range). **c** An example of DeepFRI predictions for Rosetta models of a lipid-binding protein (PDB id: 1IFC) with different TM-scores computed against its native structure. The DeepFRI output score >0.5 is considered as a significant prediction. Precision-recall curves showing the: **d** performance of our method, trained only on PDB experimental structures, and evaluated on homology models from SWISS-MODEL (red), in comparison to the CNN-based method (DeepGO) trained only on PDB sequences, and BLAST baselines are shown in blue and gray, respectively; **e** performance of DeepFRI trained on PDB (blue), SWISS-MODEL (orange) and both PDB and SWISS-MODEL (red) structures in comparison to the BLAST baseline (gray). The dot on the curve indicates where the maximum F-score is achieved (the perfect prediction should have *F*_max_ = 1 at the top right corner of the plot).

First, we explore how DeepFRI trained on PDB structures tolerates modeling errors, by comparing its performance on models obtained from SWISS-MODEL^13^ and other de novo structure prediction protocols (see Figs. 2a, d). We extract the sequences from about 700 experimentally annotated PDB chains (we refer to this dataset as PDB700), carry out structure prediction using both the Rosetta macro-molecular modeling suite^57^ and the contact predictor DMPfold^12^, and obtain the lowest energy model for each chain and method (see “Methods” section). We construct two kinds of *C*_*α*_–*C*_*α*_ contact maps for each PDB chain—one from its experimental (i.e., NATIVE) structure and one from the lowest-energy (i.e., LE) model. DeepFRI exhibits higher performance (with *F*_max_ = 0.657/0.633/0.619 for native structures and models from DMPFold and Rosetta, respectively) than that of the CNN-based method DeepGO (*F*_max_ = 0.525) even when accounting for errors in predicted contact maps (Fig. 2a). To further test the robustness in predicting GO terms with degrading quality of predicted models, we compute the *F*_max_ score on a set of Rosetta models with different template modeling scores (TM-scores)^58^ and compare them to the results from the sequence-only CNN model (see Fig. 2b). Specifically, for each sequence in the PDB700 dataset, we obtain 1500 Rosetta models with different TM-scores computed against their corresponding native structure. Even for low TM-scores we obtain better performance in GO term classification than the sequence-only CNN-based method (Fig. 2c). For example, Fig. 2c shows the output of DeepFRI with varying quality (TM-score) of Rosetta models of rat intestinal lipid-binding apoprotein (PDB id: 1IFC). For models with TM-scores >0.58, DeepFRI correctly predicts four GO terms including lipid binding (GO:0008289), whereas for a TM-score >0.73, DeepFRI correctly predicts even more specific function (i.e., fatty acid binding, GO:0005504, a child term of lipid binding). Here, we consider DeepFRI scores above 0.5 to be significant.

Even though Rosetta models often result in noisy contact maps, the performance of our method on the lowest energy models is not drastically impaired (Fig. 2a), which is due to the high denoising ability of the GCN implied by a high correlation between GCN features extracted from NATIVE and LE contact maps (see Supplementary Fig. 4). Moreover, the high tolerance for predicting functions from low-quality models is due to powerful language model features, which the model is mainly relying on when making those predictions.

Second, we examine the inclusion of homology models into the DeepFRI training procedure. A large number of diverse structures in the training set is an important prerequisite for more accurate and robust performance of our deep learning-based method. To this end, we combine ~30 k non-redundant experimental structures from the PDB and ~220 k non-redundant homology models from the SWISS-MODEL repository. Inclusion of SWISS-MODEL models not only results in more training examples and consequently in more accurate performance (*F*_max_ = 0.455/0.545 on structures from the PDB/PDB & SWISS-MODEL, see Fig. 2e), but it also results in a larger GO term coverage, especially in the number of very specific, rarely-occurring GO terms (information content, IC >10; Supplementary Fig. 5). Comparing the performance of our model with the CNN-based method, DeepGO^38^ that operates only on sequences, and the BLAST baseline, we observe that our method benefits greatly from homology models (Fig. 2e).

### DeepFRI outperforms other state-of-the-art methods

To compare the performance of our method with previously published methods, we use a test set of PDB chains with experimentally confirmed functional annotations, comprising of subsets of PDB chains with varying degrees of sequence identity to the training set. We compare our method to two sequence-based annotation transfer methods (i.e., BLAST^27^ and FunFams^24^), one state-of-the-art deep learning method (DeepGO^38^), and one feature engineering-based machine learning method (FFPred^31^). CAFA challenges commonly use the BLAST baseline, in which every test sequence receives GO terms that are transferred from the sequence in the training set with the score being the pairwise sequence identity. FunFams is one of the top-performing methods in CAFA challenges in which test sequences are scanned against a library of HMMs of CATH superfamilies. A test sequence is first mapped to a most likely FunFam (i.e., with the highest HMM score); then GO terms and EC numbers of that FunFam are transferred to the test sequence. The confidence score for each predicted GO term is computed as the annotation frequency of that GO term among the seed sequences of the FunFam^24^. DeepGO is a state-of-the-art CNN-based method trained on the same number of protein sequences as DeepFRI. DeepGO uses 1D convolution layers with varying sizes of convolutional filters to extract hierarchical features from the protein sequences (see “Methods” for the architecture details).

**Fig. 3:**
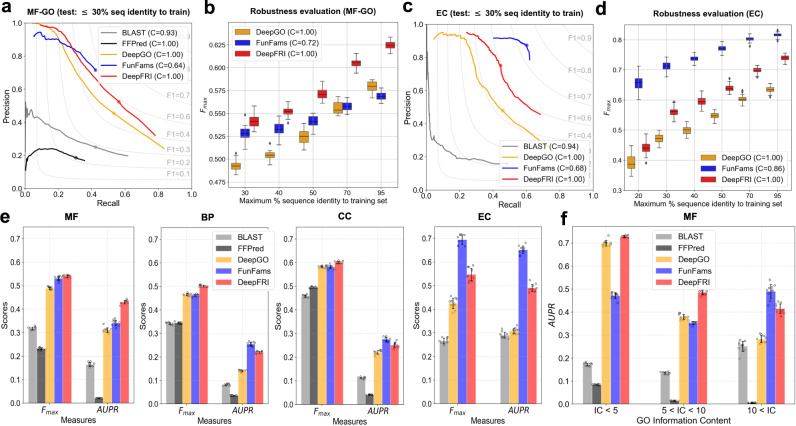
Performance over GO terms in different ontologies and EC numbers. Precision-recall curves showing the performance of different methods on (**a**) MF-GO terms and (**c**) EC numbers on the test set comprised of PDB chains chosen to have ≤30% sequence identity to the chains in the training set. Coverage of the methods is shown in the legend. Distribution of the *F*_max_ score under 100 bootstrap iterations for the top three best-performing methods applied on (**b**) MF-GO terms and (**c**) EC numbers computed on the test PDB chains and grouped by maximum % sequence identity to the training set. **e** Distribution of protein-centric *F*_max_ score and function-centric AUPR score under 10 bootstrap iterations summarized over all test proteins and GO terms/EC numbers, respectively. **f** Distribution of AUPR score on MF-GO terms of different levels of specificities under 10 bootstrap iterations. Every figure illustrates the performance of DeepFRI (red) in comparison to sequence-based annotation transfer from protein families, FunFams (blue), the CNN-based method DeepGO (orange), SVM-based method, FFPred (black), and BLAST baseline (gray). Error bars on the bar plots (**e** and **f**) represent standard deviation of the mean. In panels **b** and **d**, data are represented as boxplots with the center line representing the median, upper and lower edges of the boxes representing the interquartile range, and whiskers representing the data range (0.5 × interquartile range).

The performance of our method in comparison to state-of-the-art and baseline methods is shown in Fig. 3. In terms of both protein-centric *F*_max_, our method outperforms other methods on MF- and BP-GO terms (Fig. 3a, e). Moreover, DeepFRI learns general structure–function relationships more robustly than other methods by predicting MF-GO terms of proteins with low sequence identity to the training set. To investigate this, we partitioned our test set into groups based on maximum sequence identity to the training set and computed the protein-centric *F*_max_ score within each group (Fig. 3b). DeepFRI robustly predicts MF-GO terms of proteins with ≤30% sequence identity to the training set (with a median *F*_max_ = 0.545 compared to a median of *F*_max_ = 0.514 for FunFams and *F*_max_ = 0.491 for DeepGO), and outperforms both FunFams and DeepGO at other sequence identity cutoffs. Even though DeepFRI achieves somewhat higher precision in low recall region in predicting EC numbers at 30% sequence identity (see Fig. 3c), FunFams outperforms both DeepFRI and DeepGO with the higher *F*_max_ score across different sequence identity thresholds (Fig. 3c, d); This is especially the case for PDB chains in our test set from underrepresented protein families. However, this not the case for PDB chains belonging to protein families well represented in our training set, on which DeepFRI outperforms or has a comparable performance to FunFams (see Supplementary Fig. 19). DeepFRI outperforms the sequence-only CNN (DeepGO) and the BLAST baseline for more specific MF-GO terms (IC > 5) with fewer training examples (see Fig. 3f). In addition to testing the robustness of DeepFRI in case when a certain level of homology relationships between the training and the test set is allowed (Fig. 3b, d), we also test its robustness when the test set is comprised of non-homologous PDB chains. That is, the PDB chains belonging to protein families (i.e., Pfam^51^ IDs) and structural/fold classes (i.e., CATH^4^ IDs) different than the ones in the training set. To do this we remove PDB chains belonging to 23 largest protein families covering 3224 PDB chains from our training set, train the model on the rest, and report the results on the held our (i.e., unseen) Pfams. See Supplementary Fig. 21 for the performance results and the list of Pfam IDs in the test set. Similarly, we perform another train/test split by composing a test set of PDB chains associated with the 4 most common (and largest in our set of) folds obtained from CATH database: TIM barrel, Immunoglobulin-like, Jelly Rolls and Alpha-Beta plaits, covering in total 4759 PDB chains. We trained the model on the rest of the PDB chains, covering other structural/fold classes, and report the performance results on the test set (see Supplementary Fig. 22). In the first case, we observe higher performance of DeepFRI (*F*_max_ = 0.6) than in the second case (*F*_max_ < 0.3 across all 4 CATH folds), which can be explained by the fact that DeepFRI’s LM, pre-trained on the entire Pfam database, is helping the model generalize well on the unseen Pfams. Thus, the second case is a much more reliable setting for testing the robustness of DeepFRI. In the second case, a much lower performance of DeepFRI is observed, indicating the difficulty of DeepFRI to generalize well on the unseen fold classes. However, it can still generalize its performance on these folds better than sequence-based DeepGO and BLAST baseline indicated by the higher value of *F*_max_ score (Supplementary Fig. 22).

It is important to note that different methods encompass different subsets of the GO-term vocabulary and that a key advantage of using comparative models (for instance from SWISS-MODEL) in training is the increase in the size of the vocabulary encompassed by our method. Comparison to the standard feature engineering-based, SVM-based method FFPred, is shown in Supplementary Fig. 6. Given that FFPred is limited in the number of GO terms for which it makes predictions (131 MF-GO, 379 BP-GO, and 76 CC-GO on our test set), and also it cannot predict EC numbers, we only show the result averaged over a subset of GO terms common to all methods. Moreover, different methods have different coverages, i.e., the number of proteins in our test set for which they make predictions (see legend in Fig. 3a–d). For example, FunFams is not able to predict MF-GO terms/EC numbers for 28%/14% of proteins in our test set (the total coverage for the entire test set is shown in legends in Fig. 3b, d).

We explored the performance of our method on individual GO terms. We observe that for the majority of MF-GO terms, DeepFRI outperforms the sequence-only CNN method, indicating the importance of structural features in improving performance (see also Supplementary Fig. 7). DeepFRI outperforms the CNN on almost all GO terms with an average PDB chain length ≥400 (see Supplementary Fig. 7), illustrating the importance of encoding distant amino acid contacts via the structure graph. This demonstrates the superiority of graph convolutions over sequence convolutions in constructing more accurate protein features when key functional sites are composed of distal sequence elements (as is the case for more complex folds with higher contact order)^59^. Specifically, in the case of long protein sequences (e.g., >400 residues), a CNN with reasonable filter lengths, would most likely fail to convolve over residues at different ends of the long sequence, even after applying multiple consecutive CNN layers; whereas, graph convolutions applied on contact maps would, in 3 layers or less, access feature information from the complete structure.

### Class activation maps increase the resolution from protein-level to region-level predictions

Many proteins carry out their functions through spatially clustered sets of important residues (e.g., active sites on an enzyme, ligand-binding sites on a protein, or protein–protein interaction sites). This is particularly relevant in the Molecular Function branch of the GO hierarchy, or for EC numbers, and less so for terms encoded in the Biological Process branch. Designing ML methods for identifying such functional residues have been the subject of many recent studies^21,22,24,60^. They exploit features from sequence or structure to train classifiers on existing functional sites in order to predict new ones. Even though DeepFRI was not designed or trained explicitly to predict residue-level annotations, we show how this is achieved by post-processing methods.

**Fig. 4:**
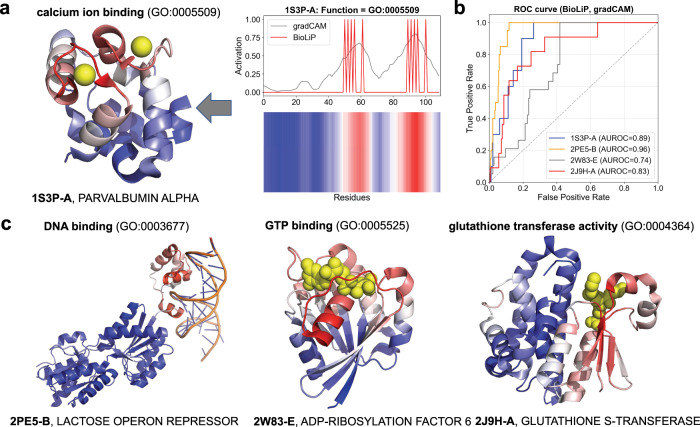
Automatic mapping of function prediction to sites on protein structures. **a** An example of the gradient-weighted class activation map for ‘Ca Ion Binding’ (right) mapped onto the 3D structure of rat *α*-parvalbumin (PDB Id: 1S3P), chain A (left), annotated with calcium ion binding. The two highest peaks in the grad-CAM activation profile correspond to calcium-binding residues. **b** ROC curves showing the overlap between grad-CAM activation profiles and binding sites, retrieved from the BioLiP database, computed for the PDB chains shown in panel (**c**). **c** Examples of other PDB chains annotated with DNA binding, GTP binding, and glutathione transferase activity. All residues are colored using a gradient color scheme matching the grad-CAM activity profile, with more salient residues highlighted in red and less salient residues highlighted in blue. No information about co-factors, active sites, or site-specificity was used during training of the model.

To better interpret decisions made by neural networks, recent work in ML has provided several new approaches for localizing signal to regions of the input feature space that lead to a given positive prediction^61–64^. In computer vision these methods determine the regions of images that lead to positive object classifications^48^; in NLP these methods identify sub-regions of documents^65^. Recent work in computer vision uses gradient-weighted Class Activation Maps (grad-CAMs) on trained CNN-based architectures to localize the most important regions in images relevant for making correct classification decisions^48^. We use grad-CAMs, adapted for post-training analysis of GCNs. For each protein, DeepFRI detects function-specific structural sites by identifying residues relevant for making accurate GO term prediction (for DeepFRI model trained on MF-GO terms), or EC prediction (for DeepFRI model trained on EC numbers). See an example of grad-CAM and its corresponding heatmap over the sequence in Fig. 4a, right. It does so by first computing the contribution of each graph convolutional feature map of the model (trained on the MF-GO dataset) to the GO term prediction, and then by summing the feature maps with positive contributions to obtain a final residue-level activation map (see “Methods”).

For site-specific MF-GO terms (i.e., GO terms describing different types of ligand binding), we provide four examples where we automatically and correctly identify functional sites for several functions where binding sites are known (see Fig. 4). Figure 4a shows the grad-CAM identified residues for a calcium ion binding (GO:0005509) of *α*-parvalbumin protein (PDB id: 1S3P). The two highest peaks in the profile correspond to the calcium-binding residues in the structure of the protein (Fig. 4a, left). Indices of the calcium-binding residues in 1S3P were retrieved from the BioLiP database^66^ and compared to the residues identified by our method by using receiver operating characteristic (ROC) curves. The ROC curve shows the relation between sensitivity or true positive rate (ratio of functional residues identified as salient) and 1-specificity or false positive rate (ratio of non-functional residues identified as non-salient). A high area under the ROC curve indicates high correspondence between annotated binding sites and our predictions, meaning high accuracies in residue-level predictions. Sample ROC curves for other functions including DNA binding (GO:0003677), GTP binding (GO:0005525), and glutathione transferase activity (GO:0004364) computed between the binary profile representing binding sites from BioLiP and the grad-CAM profile are depicted in Fig. 4b, and structural visualizations in Fig. 4c. Our study of grad-CAMs against BioLiP database reveals that the highest performing group of GO terms are related to functions with known site-specific mechanisms or site-specific underpinnings.

We depict examples (with high AUROC scores) for which grad-CAMs correctly identify binding regions in Supplementary Figs. 8–15. For various GO terms, the functional sites correspond to known binding sites or conserved functional regions (see Supplementary Figs. 8–15). Interestingly, our model is not explicitly trained to predict functional sites, but instead such predictions stem solely from the grad-CAM analysis of the graph convolution parameters of the trained model; thus, the ability of the method to correctly map functional sites supports our argument that the method is general and capable of predicting functions in a manner that transcends sequence alignment.

**Fig. 5:**
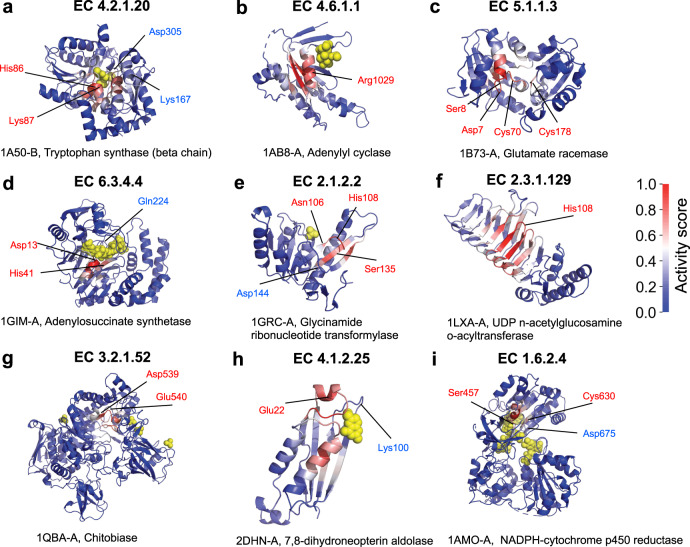
Identifying catalytic residues in enzymes using grad-CAM applied on the DeepFRI model trained on EC numbers. All residues are colored using a gradient color scheme matching the grad-CAM activity score, with more salient residues highlighted in red and less salient residues highlighted in blue. The PDB chains (shown in panels **a**–**i**) are annotated with all of its known catalytic residues (available in Catalytic Site Atlas), with a residue number and a pointer to the location on the structure. Residues correctly identified by our method are highlighted in red.

A similar approach can be used for predicting catalytic residues and active sites of proteins. Specifically, we apply grad-CAM approach on the DeepFRI model trained on EC numbers. To evaluate our predictions, we use a dataset composed of enzymes available in the Catalytic Site Atlas (CSA)^67^, a database that provides enzyme annotations specifying catalytic residues that have been experimentally validated and published in the primary literature. We use a manually curated dataset of 100 evolutionarily divergent enzymes from the CSA provided by Alterovitz et al.^60^ used for training their method ResBoost. Figure 5 shows results for a subset of PDB chains in this dataset, covering different EC numbers. Using the CSA as ground truth, we compute a ROC curve quantifying the accuracy of DeepFRI in predicting catalytic residues (see Supplementary Fig. 16). This result is not directly comparable to the performance results of ResBoost because we computed it only on a subset of 38 enzymes (out of 100 enzymes used for training ResBoost) for which EC numbers were in our training set. Moreover, DeepFRI is not designed to perform training on existing catalytic residues in the cross-validation manner (i.e., by hiding some catalytic residues in the training of the model, and then predicting on them) as ResBoost and it cannot control the trade-off between sensitivity and specificity in predicting catalytic residues. DeepFRI is also not explicitly trained to predict catalytic residues using a set of enzymes with known catalytic residues and information about their positions in the structure. Surprisingly, a high AUROC score of 0.81 (Supplementary Fig. 16) stems solely from the grad-CAM analysis of our DeepFRI model trained on EC numbers.

Performing functional site identification is also very efficient as it does not require any further training or modification of the model’s architecture. The site-specificity afforded by our function predictions is especially valuable for poorly studied, unannotated proteins. Site-specific predictions provide first insights into the correctness of predictions and frame follow-up validation experiments, for example, using genetics or mutagenesis to test site-specific predictions.

### Temporal holdout evaluation emphasizes DeepFRI’s performance in a realistic scenario

We also evaluate the performance of our method in a more realistic scenario using a temporal holdout strategy similar to the one in CAFA^27–29^. That is, we composed a test set of PDB chains by looking at the difference in GO annotations of the PDB chains in the SIFTS^56^ database between two releases separated by ~6 months—releases 18 June 2019 and 04 January 2020. We identified ~3000 PDB chains that did not have annotations in the 2019 SIFTS release and gained new annotations in the 2020 SIFTS release (see “Methods”). We evaluated the performance of DeepFRI on the newly annotated PDB chains from the 2020 SIFTS release. DeepFRI significantly outperforms both BLAST and DeepGO (see Supplementary Fig. 17). Furthermore, we highlight examples of PDB chains with correctly predicted GO terms for which both BLAST and DeepGO are failing to produce any meaningful predictions, indicating again the importance of structural information (see Supplementary Fig. 17).

### DeepFRI makes reliable predictions on unannotated PDB and SWISS-MODEL chains

A large number of high-quality protein structures in both the PDB and SWISS-MODEL lack or have incomplete functional annotations in the databases we used for training and testing our models. For example, analysis of the SIFTS June 2019 release^56^ reveals that around 20,000 non-redundant, high-quality PDB chains currently lack GO term annotations. Similarly, around 13,000 SWISS-MODEL chains lack Swiss-Prot GO term annotations. Interestingly, even though the PDB chains lack GO term annotations, many have additional site-specific functional information present in their PDB files, for instance through ligands, co-factors, metals, DNA, and RNA. We use these cases to verify their function and discuss them in depth. A set of predictions, including many for truly unknown PDB chains, is provided in Supplementary File 1. For example, there are a number of PDB chains binding metal ions that have known binding residues in BioLip^66^, but missing GO term annotations (GO:0046872). In other cases, the function, albeit missing in SIFTS, is directly implied in the name of the protein (e.g., a zinc finger protein without zinc ion binding (GO:0008270) annotation). Here, we apply our method to these unannotated PDB chains, as a part of a blind experiment, to evaluate our predictions at the chain-level and the residue-level through the grad-CAM approach. We also make predictions on SWISS-MODEL chains.

**Fig. 6:**
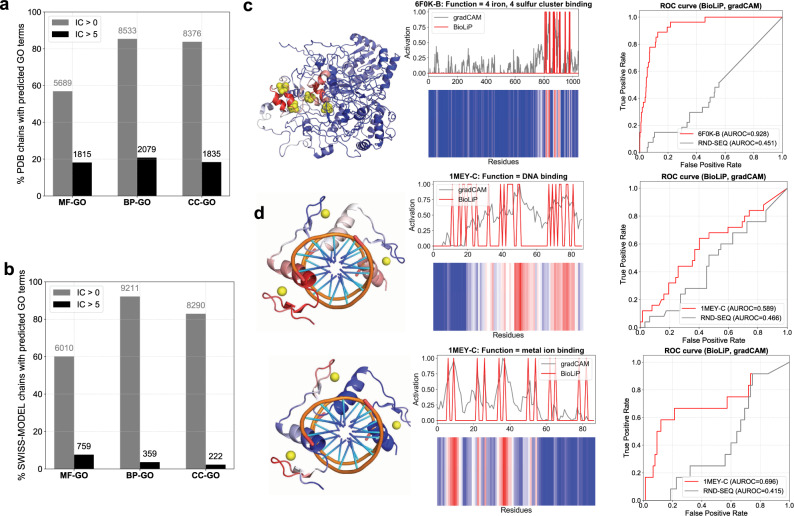
Predicting and mapping function to unannotated PDB & SWISS-MODEL chains. Percentage/number of PDB chains (**a**) and SWISS-MODEL chains (**b**) with MF-, BP-, and CC-GO terms predicted by our method; the number of specific GO term predictions (with IC >5) are shown in blue and red for PDB and SWISS-MODEL chains, respectively. **c** An example of a Fe–S-cluster-containing hydrogenase (PDB Id: 6F0K), found in Rhodothermus marinus, with missing GO term annotations in SIFTS (unannotated). The PDB chain lacks annotations in databases used for training our model and DeepFRI predicts to bind a 4Fe–4S iron–sulfur cluster with high confidence score. The predicted grad-CAM profile significantly overlaps with ligand-binding sites of 4Fe–4S obtained from BioLiP, as shown by the ROC curve. **d** grad-CAM profiles for predicted DNA binding and metal ion binding functions mapped onto the structure of an unannotated zinc finger protein (PDB Id: 1MEY) found in Escherichia coli; the corresponding ROC curves show significant overlap between the grad-CAM profile and the binding sites obtained from BioLiP.

Supplementary Data Files 1 and 2 contain all DeepFRI high-confidence predictions for the PDB and SWISS-MODEL chains. In Fig. 6a, b, we show their statistics, with the total number of PDB and SWISS-MODEL chains predicted with all and more specific (Information Content, IC >5) GO terms. Some interesting unannotated PDB chains with known ligand-binding information include 4-iron, 4-sulfur cluster binding (GO:0051539) of a Fe–S-cluster-containing hydrogenase (PDB id: 6F0K), shown in Fig. 6c. Iron–sulfur clusters are important in oxidation-reduction reactions for electron transport and DeepFRI accurately predicts their binding sites as shown by the corresponding ROC curve, computed between the predicted grad-CAM profile and the known 4Fe–4S cluster binary binding profile retrieved from BioLiP. Another example includes DNA binding (GO:0003677) and metal ion binding (GO:0046872) of the zinc finger protein (PDB Id: 1MEY) with predicted grad-CAM activity mapped onto the same structure and validated experimentally for both DNA and metal (Fig. 6d).

## Discussion

Here we describe a deep learning method for predicting protein function from both sequences and contact map representations of 3D structures. Our method DeepFRI is trained on protein structures from the PDB and SWISS-MODEL and rapidly predicts both GO terms and EC numbers of proteins and improves over state-of-the-art sequence-based methods on the majority of function terms. Features learned from protein sequences by the LSTM-LM and from contact maps by the GCN lead to substantial improvements in protein function prediction, therefore enabling novel protein function discoveries. Although high-quality sequence alignment is often sufficient to transfer folds or structural information^68^, sequence alignments are challenging to use to transfer function (as evidenced by the poor performance of the CAFA-like BLAST benchmark) due to the need for different thresholds for different functions, partial alignments, and domain structures, protein moonlighting, and neofunctionalization^27,29,69^. Thus, one important advantage of DeepFRI is its ability to make function predictions beyond homology-based transfer by extracting local sequence and global structural features^27^.

By comparing function prediction performance on DMPFold and Rosetta models and their corresponding experimentally determined structures, we demonstrate that DeepFRI has a high denoising power. Our method’s robustness to structure prediction errors indicates that it can reliably predict functions of proteins with computationally inferred structures. The ability to use protein models opens the door for characterizing many proteins lacking experimentally determined structures. Further, databases with available protein models (e.g., homology models from SWISS-MODEL^13^ and ModBase^20^) can expand the training set and improve the predictive power of the model. The more extensive use of homology models will be the subject of a future study.

While this paper mainly focuses on introducing efficient and accurate function prediction models, it also provides a means of interpreting prediction results. We demonstrate on multiple different GO terms that the DeepFRI grad-CAM identifies structurally meaningful site-specific prediction, for instance from ligand-binding sites. For some PDB chains, the accuracy of the DeepFRI grad-CAM in identifying binding residues is quite remarkable, especially since the model is not designed to predict functional residues and the ligand-binding information was not given to the model a priori. However, the main disadvantage of considering this to be a site-specific function prediction method lies in the multiple meanings of grad-CAMs. Specifically, for some GO terms related to binding, grad-CAMs do not necessarily identify binding residues/regions; instead, they identify regions that are conserved among the sequences annotated with the same function. This can be explained with the fact that any neural network, including ours, would always tend to learn the most trivial features that lead to the highest accuracy^70,71^.

In conclusion, here we describe a method that connects two key problems in computational biology, protein structure prediction and protein function prediction. Our method linking deep learning with an increasing amount of available sequence and structural data has the potential to meet the annotation challenges posed by ever-increasing volumes of genomic sequence data, offering new insights for interpreting protein biodiversity across our expanding molecular view of the tree of life.

## Methods

### Construction of contact maps

We collect 3D atomic coordinates of proteins from the Protein Data Bank (PDB)^19^. As the PDB contains extensive redundancy in terms of both sequence and structure, we remove identical and similar sequences from our set of annotated PDB chains. We create a non-redundant set by clustering all PDB chains (for which we were able to retrieve contact maps) by blastclust at 95% sequence identity (i.e., number of identical residues out of the total number of residues in the sequence alignment). Then, from each cluster we select a representative PDB chain that is annotated (i.e., has at least one GO term in at least one of the three ontologies) and which is of high quality (i.e., has a high-resolution structure). In addition to PDB structures, we also obtained homology models from the SWISS-MODEL repository^13^. We include only annotated SWISS-MODEL chains (i.e., having at least one GO term in at least one of the three GO ontologies) in our training procedure. We remove similar SWISS-MODEL sequences again at 95% sequence identity. Including SWISS-MODEL models leads to a 5-fold increase in the number of training samples (see Supplementary Table 1) and also in a larger coverage of more specific GO terms (see Supplementary Fig. 5).

To construct contact maps, we consider two resides to be in contact if the distance between their corresponding *C*_*α*_ atoms is <10 Å. We refer to this type of contact maps as CA-CA. We have also considered two other criteria for contact map construction. Two residues are in contact if (1) the distance between any of their atoms is <6.5 Å (we refer to this type of contact maps as ANY-ANY) and (2) if the distance between their Rosetta neighbor atoms is less than sum of the neighbor radii of the amino acid pair (we refer to this type of contact maps as NBR-NBR). Rosetta neighbor atoms are defined as *C*_*β*_ atoms for all amino acids except glycine where *C*_*α*_ is used. An amino acid neighbor-radius describes a potential interaction sphere that would be covered by the amino acid side chain as it samples all possible conformations. Neighbor–neighbor contact maps are therefore more indicative of side-chain–side-chain interactions than *C*_*α*_–*C*_*α*_ maps. To conserve the memory avoid training the model on protein chains with long sequences, we only construct contact maps for chains between 60 and 1000 residues. We have also experimented with different distance thresholds for CA-CA and ANY-ANY contact maps. We found that our method produced similar results when trained on these contact maps with a *C*_*α*_–*C*_*α*_ distance of 10 Å, producing slightly better results (see Supplementary Fig. 3).

### Functional annotations of PDB & SWISS-MODEL chains

For training our models we use two sets of function labels: (1) Gene Ontology (GO)^7^ terms and (2) enzyme commission (EC) numbers^72^. GO terms are hierarchically organized into three different ontologies—molecular function (MF), biological process (BP), and cellular component (CC). We train our models to predict GO terms separately for each ontology. The summary of GO identifiers as well as EC numbers for each PDB and SWISS-MODEL chain were retrieved from SIFTS^56^ (structure integration with function, taxonomy, and sequence) and UniProt Knowledgebase databases, respectively.

SIFTS transfers annotations to PDB chains via residue-level mapping between UniProtKB and PDB entries. All the annotation files were retrieved from the SIFTS database (2019/06/18) with PDB release 24.19 and UniPortKB release 2019.06. We consider annotations that are (1) not electronically inferred (in figure captions/legends, we refer to those as EXP), specifically, we consider GO terms with the following evidence codes: EXP (inferred from experiment), IDA (inferred from direct assay), IPI (inferred from physical interaction), IMP (inferred from mutant phenotype), IGI (inferred from genetic interaction), IEP (inferred from expression pattern), TAS (traceable author statement), and IC (inferred by curator), and (2) electronically inferred (in figure captions/legends, we refer to those as IEA—inferred from electronic annotation). Furthermore, we focus only on specific MF-, BP-, and CC-GO terms that have enough training examples from the non-redundant training set (see the section above). That is, we select only GO terms that annotate >50 non-redundant PDB/SWISS-MODEL chains. We retrieved enzyme classes for sequences and PDB structures from the levels 3 and 4 (most specific levels) of the EC tree. The number of GO terms and EC classes in each ontology is represented in Supplementary Table 1.

In our analyses, we differentiate GO terms based on their specificity, expressed as Shannon information content (IC)^73^:

1 $${\mathrm{IC}}({\mathrm{G}}{{\mathrm{O}}}_{i})=-{\mathrm{log}}_{2}{\mathrm{Prob}}({\mathrm{G}}{{\mathrm{O}}}_{i}),$$

where, Prob(GO_*i*_) is the probability of observing GO term *i* in the UniProt-GOA database (*n*_*i*_/*n*, where *n*_*i*_—number of proteins annotated with GO term *i* and *n*—total number of proteins in UniProt-GOA). Infrequent GO terms (i.e., more specific) have higher IC values.

### Training and test set construction

We partition the non-redundant set composed of PDB and SWISS-MODEL sequences into training, validation, and test sets, with approximate ratios 80/10/10%. The test set, comprising of only experimentally determined PDB structures and experimentally determined annotations is chosen to have PDB chains with varying degrees of sequence identity (i.e., 30%, 40%, 50%, 70%, and 95% sequence identity) to the training set. Furthermore, each PDB chain in the test set is chosen to have at least one experimentally confirmed GO term in each branch of GO. See Supplementary Table 2.

We use the CD-HIT clustering tool^74^ to select SWISS-MODEL sequences that are dissimilar to the test set and to split them into training and validation sets. We examine the performance of our method when trained only on PDB, only on SWISS-MODEL and both PDB & SWISS-MODEL contact maps; we also investigate training on only EXP and both EXP & IEA function labels (see Supplementary Fig. 18A). In all our experiments we trained the model using both EXP and IEA GO annotations), but the test set, composed of only experimentally annotated PDB chains (EXP), is always kept fixed. See Supplementary Table 1. The final results are averaged over 100 bootstraps of the test set, in all our experiments.

### Preparation of a benchmark set of protein models

The initial set of benchmark structures used here was Jane and Dave Richardson’s Top 500 dataset^75^. It is a set of hand curated, high-resolution, and high quality (the top 500 best), protein structures that were chosen for their fit to their completeness, how well they fit the experimental data, and lack of high energy structural outliers (bond angle and bond length deviations^76^). This set has been used in the past for fitting Rosetta energy/score terms and numerous other structural-bioinformatics validation tasks. Unfortunately, the structures in this set lacked sufficient annotations (many of these structures were the results of structural genomics efforts and had no, or only high level, annotations in GO and EC). Accordingly, we choose an additional 200 sequences from the PDB. These additional high-quality benchmark structures were chosen by taking 119K chains with functional annotations and filtering them with the PISCES Protein Sequence Culling Server^77^ with the following criteria: sequence percentage identity: ≤25, resolution: 0.0–2.0, R-factor: 0.2, sequence length: 40–500, non-X-ray entries: Excluded, CA-only entries: Excluded, Cull PDB by chain.

This left us with 1606 SIFTS annotated chains from which we randomly selected 200 chains. These PDB chains together with the Top500 PDB chains (we refer to this combined set as PDB700) were then excluded from all phases of model training. The performance of our method on this set of PDB chains is shown in Fig. 2a. In Supplementary Fig. 4, we demonstrate the denoising capabilities of our method on this set of structures.

### Comparison with existing methods

#### CNNs

CNNs have shown tremendous success in extracting information from sequence data and making highly accurate predictive models. Their success can be attributed to convolutional layers with a highly reduced number of learnable parameters, which allow multi-level and hierarchical feature extraction. In the last few years, a large body of work has been published covering various applications of CNNs, such as the prediction of protein functions^38^ and subcellular localization^78^, prediction of effects of noncoding-variants^79^ and protein fold recognition^80^. Here we use the CNN-based DeepGO tool^38^ in our comparison study. We describe this architecture in more detail in the Supplementary Material.

We represent a protein sequence with *L* amino acid residues as a feature matrix **X** = [**x**_1_, …, **x**_*L*_] ∈ {0, 1}^*L*×*c*^, where *c* = 26 dimensions (20 standard, 5 non-standard amino acids, and the gap symbol) are used as a one-hot indicator, **x**_*i*_ ∈ {0, 1}^*c*^, of the amino acid residue at position *i* in the sequence. This representation is fed into a convolution layer, which applies a one-dimensional convolution operation with a specified number of kernels (weight matrices or filters), *f*_*n*_, of certain length, *f*_*l*_. The output is then transformed by the rectified linear activation function (ReLU), which sets values below 0 to 0, i.e., ReLU(*x*) = max(*x*, 0). This is followed by a global max-pooling layer and a fully connected layer with sigmoid activation function for predicting probabilities of GO terms or EC enzyme classes.

In the first convolution layer, we use 16 CNN layers with *f*_*n*_ = 512 filters of different lengths (see Supplementary Material). After concatenating the outputs of the CNN layers, we obtain an *L* × 8192 dimensional feature map for each sequence. Using filters of variable lengths ensures the extraction of complementary information from protein sequences. The second layer has ∣*GO*∣ number of units for GO terms (or ∣*EC*∣ for EC) classification.

#### BLAST

BLAST baseline is used in the same way as described in CAFA1^27^: a sequence in our test set receives GO/EC annotations from all annotated sequences in our training set (comprised of SWISS-PROT sequences) with the prediction scores equal to the sequence identity scores (divided by 100) between the test and the training sequences. Prior to this, we remove all sequences from our training set that are similar to our test sequences using an E-value threshold of 1e−3, to prevent annotation transfer from homologous sequences, as previously described by Cozzetto et al.^31^.

#### FFPred

FFPred is a support vector machine (SVM)-based classifier on manually designed features derived from sequences such as transmembrane regions, secondary structures, and sequence motifs^31^.

#### FunFam

FunFam is a domain-based method that uses functional classification of CATH superfamilies for annotation transfer. The method takes each sequence and scans it against CATH FunFams using HMMER3^81^. Then it transfers all GO terms/EC numbers from the FunFams with the highest HMM score to the test sequence. We followed the procedure described here https://github.com/UCLOrengoGroup/cath-tools-genomescan to obtain GO terms and EC numbers for our test sequences. The GO term assignment score is computed as frequency of the GO terms among the seed sequences of the matched FunFam and propagated up the GO hierarchy as described in Das et al.^24^.

### LSTM language model for learning residue-level features

We use an approach similar to Bepler and Berger^35^ for training our language model. We train a LSTM language model on ~10 M sequences sampled from the entire set of sequences from Pfam^51^. The sequences are represented using 1-hot encoding (see above). The language model architecture is comprised of two stacked forward LSTM layers with 512 units each (see Fig. 1). The LSTM-LM model is trained for 5 epochs using an ADAM optimizer^82^ with a learning rate 0.001 and a batch size of 128. All hyper-parameters are determined through a grid search based on the model’s performance on the validation set.

The residue-level features, extracted from the final LSTM layer’s hidden states, **H**^*LM*^, are combined with 1-hot representation of sequences, **X**, through learnable non-linear mapping:

2 $${\bf{H}}^{{\mathrm{input}}}={\mathrm{ReLU}}({\bf{H}}^{LM}{\bf{W}}^{LM}+{\bf{XW}}^{X}+{\bf{b}})$$

where, **H**^input^ is the final residue-level feature representation passed to the fist GCN layer, **H**^(0)^ = **H**^input^ (see the equation below). We refer to this stage of our method as a feature extraction stage. The parameters, **W**^*LM*^, **W**^*X*^, and **b** are trained with the parameters of the GCN. All the parameters of the LSTM-LM are frozen during the training. We choose this strategy because it more efficient (i.e., instead of fine tuning the huge number of the LSTM-LM parameters together with GCN parameters, we only tune, **W**^*LM*^, **W**^*X*^, and **b** parameters while keeping the parameters of the LSTM-LM fixed).

### Graph convolutional network

GCNs have proven to be powerful for extracting features from data that are naturally represented as one or more graphs^42^. Here we experiment with the notion that GCNs are a suitable method for extracting features from proteins by taking into account their graph-based structure of inter-connected residues, represented by contact maps. We propose our model based on the work of Kipf and Welling^44^. A protein graph can be represented by a contact map, ${\bf{A}}\in {{\mathbb{R}}}^{L\times L}$, encoding connections between its *L* residues, and a residue-level feature matrix, ${\bf{X}}\in {{\mathbb{R}}}^{L\times c}$.

We explore different residue-level feature representations including one-hot encoding of residues as in the CNN (*c* = 26), LSTM language model (*c* = 512, i.e., the output of the LSTM layers), and no sequence features (to be able to run the GCN, in this case, the feature matrix is substituted with a diagonal identity matrix, i.e., **X** = **I**_*L*_).

The graph convolution takes both the adjacency matrix **A** and the residue-level embeddings from the previous layer, ${{\bf{H}}}^{(l)}\in {{\mathbb{R}}}^{L\times {c}_{l}}$ and outputs the residue-level embeddings in the next layer, ${{\bf{H}}}^{(l+1)}\in {{\mathbb{R}}}^{L\times {c}_{l+1}}$:

3 $${{\bf{H}}}^{l+1}=GC({\bf{A}},{{\bf{H}}}^{l})$$

where, **H**^(0)^ = **H**^input^, and *c*_*l*_ and *c*_*l*+1_ are residue embedding dimensions for layers *l* and *l* + 1, respectively. Concretely, we use the formulation of Kipf and Welling^44^:

4 $${{\bf{H}}}^{l+1}={\mathrm{ReLU}}({\widetilde{{\bf{D}}}}^{-0.5}\widetilde{{\bf{A}}}{\widetilde{{\bf{D}}}}^{-0.5}{{\bf{H}}}^{(l)}{{\bf{W}}}^{(l)})$$

where $\widetilde{{\bf{A}}}={\bf{A}}+{{\bf{I}}}_{L}$ is the adjacency matrix with added self-connections represented by the identity matrix ${{\bf{I}}}_{L}\in {{\mathbb{R}}}^{L\times L}$, $\widetilde{{\bf{D}}}$ is the diagonal degree matrix with entries ${{\bf{D}}}_{ii}=\mathop{\sum }\nolimits_{j = 1}^{L}{\widetilde{{\bf{A}}}}_{ij}$, and ${{\bf{W}}}^{(l)}\in {{\mathbb{R}}}^{{c}_{l}\times {c}_{l+1}}$ is a trainable weight matrix for layer *l* + 1.

To normalize residue features after each convolutional layer the adjacency matrix is first symmetrically normalized, hence the term ${\widetilde{{\bf{D}}}}^{-0.5}\widetilde{{\bf{A}}}{\widetilde{{\bf{D}}}}^{-0.5}$. Equation (4) updates features of each residue by a weighted sum of features of the directly connected residues in the graph (adding self-connections ensures that the residue’s own features are also included in the sum).

We also explore other types of graph convolutional layers previously proposed in the machine learning literature. Specifically, we tested the performance of DeepFRI on all of the branches of GO as well as EC classes with SAmple and aggreGatE convolutions (SAGEConv)^53^, Chebyshev spectral graph convolutions (ChebConv)^52^, Graph Attention (GAT)^54^, and a combination of different graph convolutions with different propagation rules (MultiGraphConv)^55^ in comparison to the plain Kipf & Welling graph Convolution (GraphConv)^44^. These convolutions differ in the way the features of the neighboring residues are aggregated. The performance of DeepFRI in predicting MF-GO and EC labels with these graph convolution layers is shown in Supplementary Fig. 1.

Given that we are classifying individual protein graphs with different number of residues, we use several layers, *N*_*l*_ = 3, of graph convolutions. The final protein representation is obtained by first concatenating features from all layers into a single feature matrix, i.e., ${\bf{H}}=[{{\bf{H}}}^{(1)},\ldots ,{{\bf{H}}}^{({N}_{l})}]\in {{\mathbb{R}}}^{L\times \mathop{\sum }\nolimits_{l = 1}^{L}{c}_{l}}$ and then by performing a global pooling layer after which we obtain a fixed vector representation of a protein structure, ${{\bf{h}}}^{{\mathrm{pool}}}\in {{\mathbb{R}}}^{\mathop{\sum }\nolimits_{l = 1}^{L}{c}_{l}}$. The global pooling is obtained by a sum operator over *L* residues:

5 $${{\bf{h}}}^{{\mathrm{pool}}}=\mathop{\sum }\limits_{i=1}^{L}{{\bf{H}}}_{i:}$$

We then use a fully connected layer with a ReLU activation function for computing the hidden representation from the pooled representation. This is then followed by a fully connected layer which is used for mapping the hidden representation from the previous layer to a ∣*GO*∣*x*2 output; that is, two activations for each GO term. These activations are transformed by a softmax activation function, outputting the positive and negative probability for each GO term/EC number (i.e., the final layer outputs probability vector $\hat{{\bf{y}}}$ of dimension ∣*GO*∣ × 2 (∣*EC*∣ × 2 for EC numbers) for predicting positive and negative probabilities of GO terms (EC numbers).

### Model training and hyper-parameter tuning

To account for imbalanced labels, both the CNN and GCN are trained to minimize the weighted binary cross-entropy cost function that gives higher weights to the GO term with fewer training examples:

6 $${\mathcal{L}}({\boldsymbol{\Theta }})=-\frac{1}{N}\mathop{\sum }\limits_{i=1}^{N}\mathop{\sum }\limits_{j=1}^{| GO| }\mathop{\sum }\limits_{k=1}^{2}{w}_{j}{y}_{ijk}{\mathrm{log}}\,({\hat{y}}_{ijk})$$

where **Θ** is the set of all parameters in all layers to be learned; ${w}_{j}=\frac{N}{{N}_{j}^{+}}$ is the class weight for function *j*, with ${N}_{j}^{+}$ being the number of positive examples associated with function *j*; *N* is the total number of samples and ∣*GO*∣ is the total number of functions (i.e., GO terms); *y*_*ijk*_ is the true binary indicator for sample *i* and function *j* (i.e., *y*_*ij*1_ = 1, if sample *i* is annotated with function *j*, and *y*_*ij*2_ = 0, otherwise) and ${\hat{y}}_{ij1}$ is the predicted probability that sample *i* is annotated with function *j*. In the inference phase, we say we predict GO terms/EC numbers if the positive probability is >0.5.

All hyper-parameters are determined through a grid search based on the model’s performance on the validation set. The validation set is comprised of ~10% randomly chosen samples from the training set. To avoid overfitting, we use an early stopping criterion with *patience* = 5 (i.e., we stop training if the validation loss does not improve in 5 epochs). We use the ADAM optimizer^82^ with a learning rate *lr* = 0.0001, *β*_1_ = 0.95, and *β*_2_ = 0.95 and a batch size of 64. The default number of epochs is 200. Both GCN and CNN are implemented to deal with variable length sequences, by performing sequence/contact map padding. The entire method is implemented using the Tensorflow/Keras deep learning library (see Supplementary Note).

### Temporal holdout validation

We also evaluate the performance of our method by using temporal holdout validation similar to CAFA^27^. The temporal holdout approach ensures a more “realistic” scenario where function predictions are evaluated based on recent experimental annotations^34^. We used GO annotations retrieved from SIFTS^56^ from two time points, version 2019/06/18 (we refer to this as SIFTS-2019) and version 2020/01/04 (we refer to this as SIFTS-2020), to construct our temporal holdout test set. We form the test set from the PDB chains that did not have any annotations in SIFTS-2019 but gained annotations in SIFTS-2020. To increase the GO term coverage, we focus on the PDB chains with both EXP and IEA evidence codes. We obtain 4072 PDB chains (out of which 3115 have sequences <1200 residues). We use our model (trained on SIFTS-2019 GO annotations) to predict functions of these newly annotated PDB chains. We evaluate our predictions against the annotations from SIFTS-2020. The results for MF-, BP-, and CC-GO terms are shown in Supplementary Fig. 17. We also show a few examples of the PDB chains with correctly predicted MF-GO terms by our method, for which both BLAST and DeepGO are not able to make any significant predictions.

### Residue-level annotations

We use a method based on Gradient-weighted Class Activation Map (grad-CAM)^48^ to localize function predictions on a protein structure (i.e., to find residues with highest contribution to a specific function). Grad-CAM is a class-discriminative localization technique that provides visual explanations for predictions made by CNN-based models. Motivated by its success in image analysis, we use grad-CAM to identify residues in a protein structure that are important for the prediction of a particular function.

In grad-CAM, we first compute the contribution of each filter, *k*, in the last convolutional layer to the prediction of function label *l* by taking the derivative of the output of the model for function *l*, *y*^*l*^, with respect to feature map ${{\bf{F}}}_{k}\in {{\mathbb{R}}}^{L}$ over the whole sequence of length *L*:

7 $${w}_{k}^{l}=\mathop{\sum }\limits_{i=1}^{L}\frac{\partial {y}^{l}}{\partial {F}_{k,i}}$$

where ${w}_{k}^{l}$ represents the importance of feature map *k* for predicting function *l*, obtained by summing the contribution from each individual residue. Finally, we obtain the function-specific heatmap in a residue space by making the weighted sum over all feature maps in the last convolutional layer:

8 $${\mathrm{CA{M}}}^{l}[i]={\mathrm{ReLU}}\left(\mathop{\sum}\limits_{k}{w}_{k}^{l}{F}_{k,l}\right)$$

where the ReLU function ensures that only features with positive influence on the functional label are preserved; CAM^*l*^[*i*] indicates the relative importance of residue *i* to function *l*. The advantage of grad-CAM is that it does not require re-training or changes in the architecture of the model which makes is computationally efficient and directly applicable to our models. See Supplementary Figs. 8–15 representing grad-CAM mapped onto 3D structure of PDB chains with known ligand-binding information and Fig. 4 for grad-CAM mapped to 3D structure of PDB chains with known active sites.

Residue-level evaluation: for each individual protein and its predicted MF-GO term/EC number, we measure the ability of our method in predicting binding or active sites. This measure can only be computed for the minority of proteins with detailed site-specific annotations; here we rely on the site-specific annotation available in the BioLiP database^66^ for ligand-binding proteins and the Catalytic Site Atlas (CSA)^67^ for enzymes.

For example, for a given protein of *L* residues, we construct a ligand-binding binary profile (retrieved from BioLiP), **s** ∈ {0, 1}^*L*^, indicating residues known to bind a specific ligand (e.g., ATP); i.e., *s*_*i*_ = 1 if residue *i* is a ligand-binding residue, *s*_*i*_ = 0 otherwise. For the same protein and its corresponding predicted function (e.g., ATP binding (GO:0005524)), we compute a real-valued grad-CAM profile from our pre-trained DeepFRI method, $\hat{{\bf{s}}}\in {[0,1]}^{L}$, indicating the functional importance of each residue. To show how well the grad-CAM profile recovers known binding sites, we compute the area under the ROC curve, representing the values of sensitivity for a given 1-specificity (false positive rate), using the sliding threshold approach; we then compute the area under the ROC curve (AUROC) using the trapezoid rule^83^. See Supplementary Figs. 8–15 for examples of ROC curves for different MF-GO terms and Supplementary Fig. 16 for ROC curve showing aggregate performance over different EC numbers.

### Reporting summary

Further information on research design is available in the Nature Research Reporting Summary linked to this article.

## Supplementary information

Supplementary Information

Description of Additional Supplementary Files

Supplementary Data 1

Supplementary Data 2

Reporting Summary

## Data Availability

Our training, validation, and test data splits are available from our github page at https://github.com/flatironinstitute/DeepFRI. All other relevant data are available from the authors upon reasonable request. Source data are provided with this paper.

## Source data

Source Data

## Code availability

The source code for training the DeepFRI model, together with neural network weights are available for research and non-commercial use at https://github.com/flatironinstitute/DeepFRI and it can be cited by using 10.5281/zenodo.4650027. A web service of our method is available at https://beta.deepfri.flatironinstitute.org/.
