# Supplementary material for: Structure-based protein function prediction using graph convolutional networks
Source: Nat Commun. 2021 May 26;12:3168. doi: 10.1038/s41467-021-23303-9 (PMC8155034; doi:10.1038/s41467-021-23303-9)
Supplement: Supplementary file 1 — Supplementary Information [file 41467_2021_23303_MOESM1_ESM.pdf]

# Supplementary Information for

## Structure-Based Protein Function Prediction using Graph Convolutional Networks

Vladimir Gligorijevic, P. Douglas Renfrew, Tomasz Kosciolek, Julia Koehler Leman, Daniel Berenberg, Tommi Vatanen, Chris Chandler, Bryn Taylor, Ian M. Fisk, Hera Vlamakis, Ramnik J. Xavier, Rob Knight, Kyunghyun Cho & Richard Bonneau

Correspondence to: vgligorijevic@flatironinstitute.org, rb133@nyu.edu,

**This PDF file includes:**

Model architectures
Supplementary Figs. 1 to 22
Supplementary Tables 1 & 2

**Supplementary Note**

CNN architecture (using *Keras*[1] syntax)

**Input:** 1-hot encoding of sequence: (n_samples, L, 26).

- `1D CNN layer x 16 (# filters = {512, 512, 512, 512, 512, 512, 512, 512, 512, 512, 512, 512, 512, 512, 512, 512}, length = {8, 16, 25, 32, 40, 48, 56, 64, 72, 80, 88, 96, 104, 112, 120, 128}, l2_reg = 2e-4)`
- `Concatenate layer`
- `BatchNormalization`
- `Activation (ReLU)`
- `Dropout (0.3)`
- `GlobalMaxPooling`
- `Dropout (0.6)`
- `Dense (dim = |GO| or dim = |EC|)`
- `Activation (sigmoid)`

Optimization: loss = `binary_crossentropy`; optimizer = `Adam` (lr = 0.0005, β1 = 0.95, β2 = 0.99); `batch_size` = 64; `epochs` = 100; `EarlyStopping` (patience=5).

LSTM-LM architecture

**Input:** 1-hot encoding of sequence: (n_samples, L, 26).

- `LSTM (dim = 512, return_sequences=True, kernel_constrain=MinMax(-2.0, 2.0), recurrent_constrain=MinMax(-2.0, 2.0))`
- `LSTM (dim = 512, return_sequences=True, kernel_constrain=MinMax(-2.0, 2.0), recurrent_constrain=MinMax(-2.0, 2.0))`
- `TimeDistributed(Dense(26))`
- `Activation (softmax)`

Optimization: loss = `categorical_crossentropy`; optimizer = `Adam` (lr = 0.001, β1 = 0.99, β2 = 0.99); `batch_size` = 128; `epochs` = 5.

GCN architecture

**Input:** 1-hot encoding of sequence, S = (n_samples, L, 256); Normalized contact maps, A = (n_samples, L, L); Pre-trained LSTM-LM.

- `Sequence embedding:`
  - `SeqEmbedding_1 = Dense (dim = 128, use_bias=False)(S)`
  - `SeqEmbedding_2 = Dense (dim = 128, use_bias=False)(LSTM-LM(S))`

[1] https://keras.io/

```
    –  Add()(SeqEmbedding_1, SeqEmbedding_2)
    –  Activation (ReLU)
•  GCN layer (dim = 256, use_bias=False, l2_reg = 2e-4)
•  Activation (ReLU)
•  GCN layer (dim = 256, use_bias=False, l2_reg = 2e-4)
•  Activation (ReLU)
•  GCN layer (dim = 512, use_bias=False, l2_reg = 2e-4)
•  Activation (ReLU)
•  Concatenate layer (all GCN layers)
•  GlobalSumPooling
•  Dropout(0.3)
•  Dense (dim = 1024)
•  Activation (ReLU)
•  |GO| X Dense (dim = 2)
•  Activation (softmax)
```

Optimization: loss = `categorical_crossentropy`; optimizer = `Adam` (lr = 0.001, β1 = 0.99, β2 = 0.99); `batch_size` = 64; `epochs` = 200; `EarlyStopping` (patience=5).

**Evaluation metrics**

We evaluate the performance of our method using both *protein-level* and *residue-level* metrics as follows:

1) *Protein-level evaluation:* we measure the performance of our method in a) predicting functions for a particular protein (*protein-centric*) and b) predicting proteins associated with a particular GO/EC term (*term-centric*). To this end, we use two measures first proposed in CAFA[14]:

   a) *Protein*-centric F-max obtained by finding the maximum of $F_1$ score over thresholds $t \in [0,1]$:

$$F_{max} = \max_t \left\{ \frac{2 \cdot AvgPr(t) \cdot AvgRc(t)}{AvgPr(t) + AvgRc(t)} \right\} \qquad \textit{Supplementary Equation 1}$$

   where the precision is averaged over all proteins, *m(t)*, for which we predict at least one term: $AvgPr(t) = \frac{1}{m(t)} \sum_{i=1}^{m(t)} pr_i(t)$ , whereas recall is averaged over all proteins, *n*: $AvgRc(t) = \frac{1}{n} \sum_{i=1}^{n} rc_i(t)$. For a given target protein, *i*, and some value of threshold $t \in [0,1]$, the precision and recall are computed as:

$$pr_i(t) = \frac{\sum_f I(f \in P_i(t) \wedge f \in T_i)}{\sum_f I(f \in P_i(t))} \qquad \textit{Supplementary Equation 2}$$

$$rc_i(t) = \frac{\sum_f I(f \in P_i(t) \wedge f \in T_i)}{\sum_f I(f \in T_i)} \qquad \textit{Supplementary Equation 3}$$

where *f* is a GO/EC term, $T_i$ is a set of known GO/EC terms for protein *i* (for MF-GO, BP-GO and CC-GO we propagated annotations up to the root term), and $P_i(t)$ is a set of predicted GO/EC terms with score ≥ t, $I(\cdot)$ – the indicator function.

b) *Term-centric* area under the Precision-Recall curve (AUPR), where precision and recall for each term *f* are computed as:

$$pr_f(t) = \frac{\sum_i I(f \in P_i(t) \wedge f \in T_i)}{\sum_i I(f \in P_i(t))}$$ *Supplementary Equation 4*

$$rc_f(t) = \frac{\sum_i I(f \in P_i(t) \wedge f \in T_i)}{\sum_i I(f \in T_i)}$$ *Supplementary Equation 5*

For each term, *f*, we compute PR curve using the sliding window method (i.e., across all threshold values of $t \in [0,1]$) and then we compute AUPR using the trapezoid rule. Even though in same cases we report AUPR values for each individual GO term (e.g., **Supplementary Fig. 7**), in most cases we report the AUPR performance under *micro*- and *macro*- averaging; the *micro*-averaged PR curve is computed by first vectorizing the protein–function predicted scores and known binary annotations (i.e. flattening protein-function matrices), and then computing the PR curve using the sliding window method (e.g., **Fig. 2A**, **B**). The area under the PR curve, obtained by applying trapezoid rule, is known as *micro*-AUPR. *macro*-AUPR (*macro*-AUPR) is computed by first computing the AUPR for each function separately, and then averaging these values across all functions.

**Supplementary Figure 1**. Performance of our method on MF-GO terms (A) and EC numbers (B) with different Graph Convolutional layers. Distribution of protein-centric $F_{max}$ score and function-centric AUPR score under 10 bootstrap iterations. Error bars represent standard deviation of the mean.

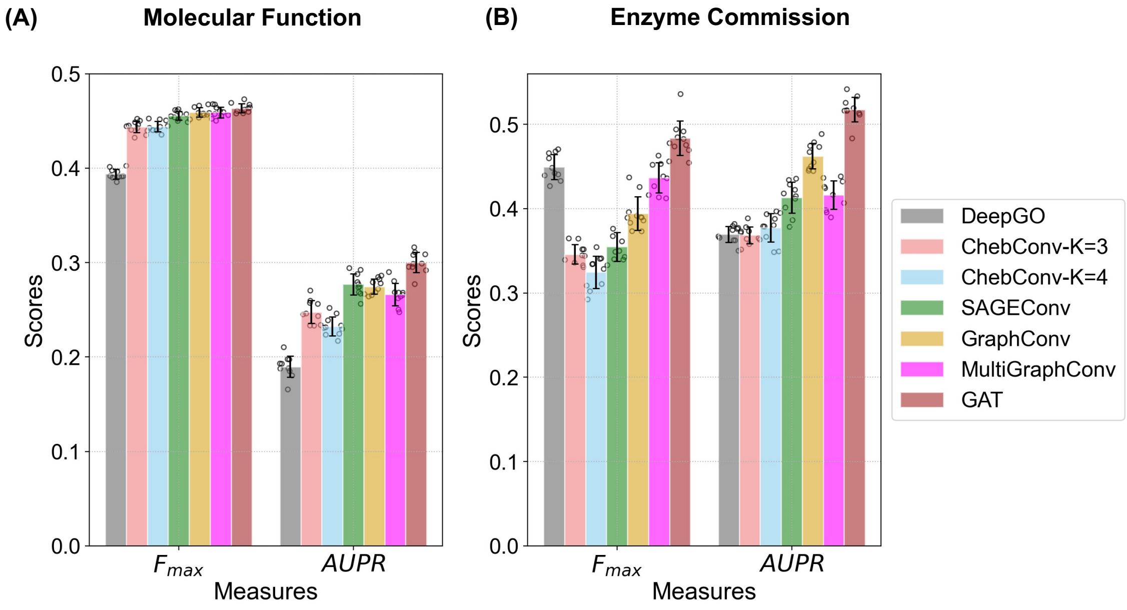


**Supplementary Figure 2**. Precision-recall curves showing the performance of different *DeepFRI* baseline architectures, in comparison to the sequence-only *CNN*, demonstrating the importance of both stages of *DeepFRI* shown in **Fig. 1** (main manuscript). The *DeepFRI* model with deep neural network architecture instead of graph convolutions is trained only on Language Model features (*DNN-LM*), with simple one-hot encoding sequence features instead of Language Model features (*GCN-1HOT*), and with both graph convolutions and Language Model features (*GCN-LM*).

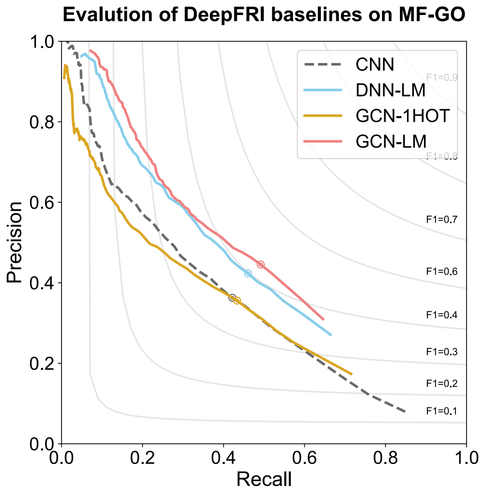

**Supplementary Figure 3.** Performance of our method in comparison to state-of-the-art CNN (*DeepGO*) and *BLAST* baseline trained on different contact maps. The model is trained on proteins with experimental (EXP) MF-GO annotations. The results are averaged over 100 bootstraps of the test set.

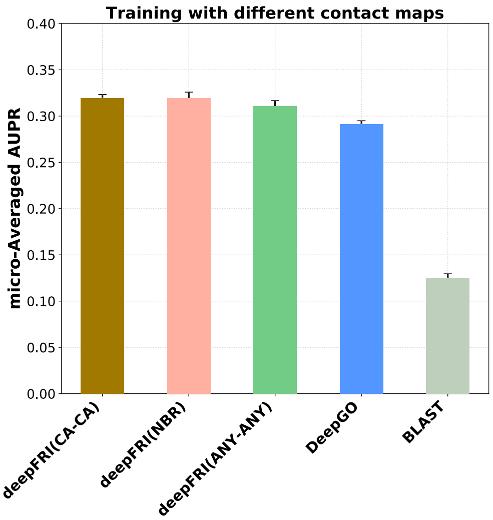


**Supplementary Figure 4**. De-noising contact maps from Rosetta models. Here we plot features from using NATIVE contact maps as input vs. the features derived from using Rosetta-predicted contact maps of Rosetta models. We show these features for the 1st, 2nd and 3rd layer of graph convolution, and note that the features extracted from the third layer of the GCN exhibit strong NATIVE-Predicted correlations, providing strong evidence that our model is tolerant to even significant error in structure predictions, and can effectively denoise structure predictions.

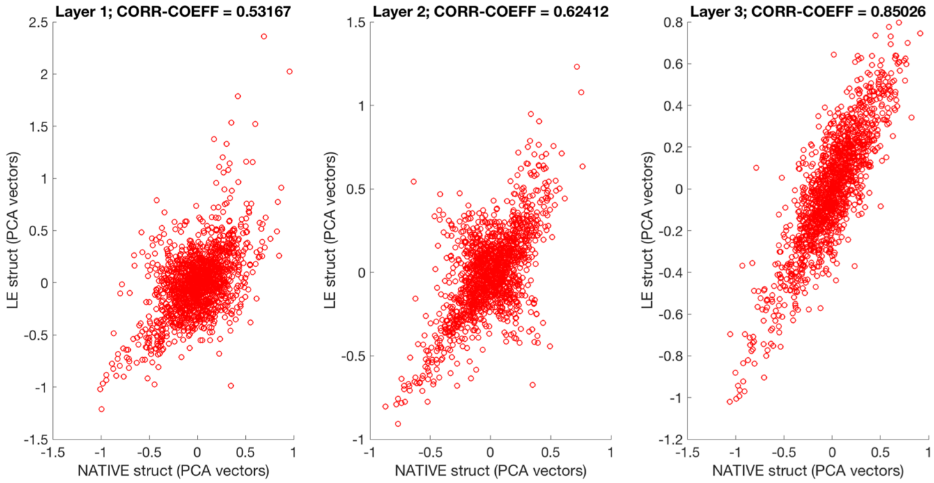

**Supplementary Figure 5**. We stratify MF-, BP- and CC-GO terms into different groups based on their specificity, expressed as Information Content (IC), see methods, and show the number of each term in each category encompassed by both the PDB-only-trained (blue) and the PDB-and-SWISS-MODEL trained DeepFRI models (red).

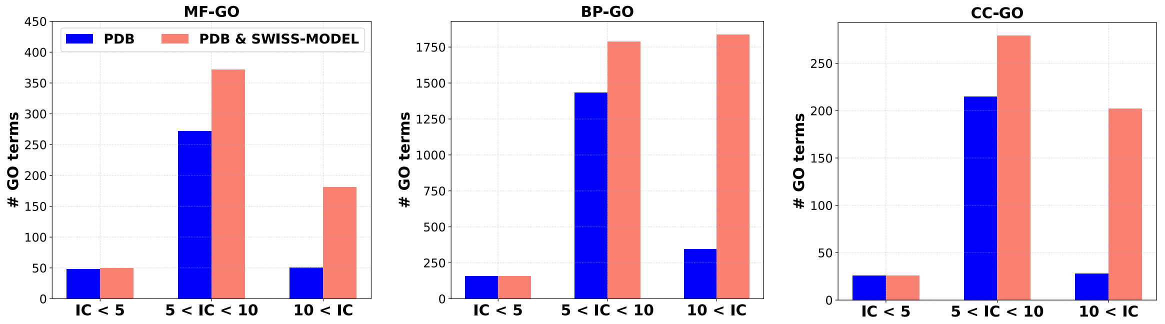


**Supplementary Figure 6**. (A) F-max and AUPR scores, summarized over all proteins and GO terms, respectively, computed on the test set comprised of PDB and SWISS-MODEL chains chosen to have < 30 % sequence identity to the sequences in the training set. The numbers in brackets indicate the number of GO terms in different ontologies that are common to all four methods; asterisks indicate where the performance of *DeepFRI* is significantly better than *DeepGOPlus* (two-sided Wilcoxon rank-sum is used to compute significance with 3 asterisks indicating pval < 0.001). The F-max and AUPR scores averaged over 100 bootstraps of the test set. Error bars represent standard deviation of the mean. (B) Micro-average precision-recall curves for each method for MF-GO terms. The curves are averaged over100 bootstraps of the test set. Error bars represent standard deviation of the mean

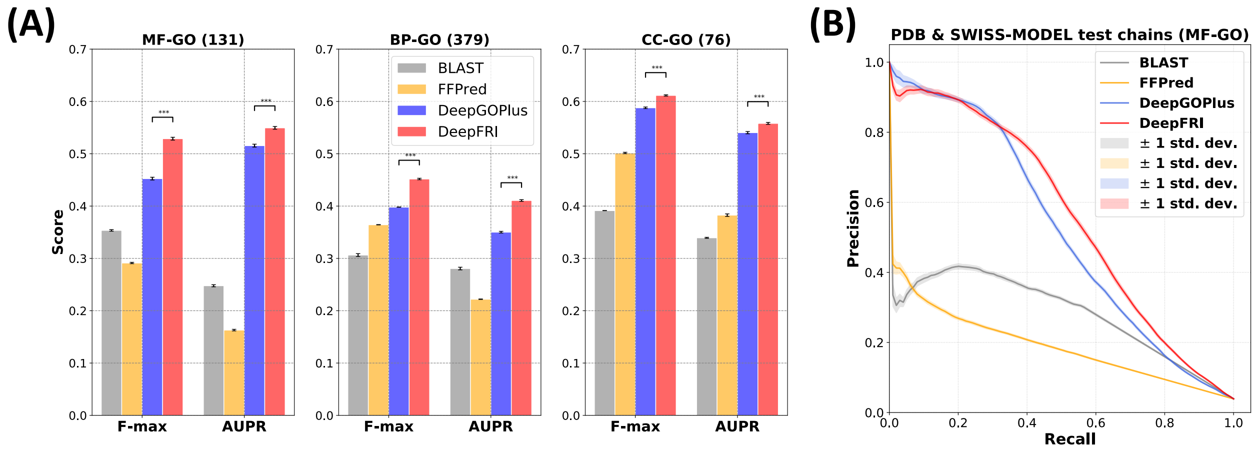

**Supplementary Figure 7**. (A) Experiments on experimentally annotated PDB chains show a difference in performance of our method vs. the CNN in predicting individual MF-GO terms. For each MF-GO term we show the *DeepFRI* and *DeepGOPlus* performance measured by the AUPR averaged over 100 bootstraps of the test proteins. Error bars represent standard deviation of the mean. Two-sided Wilcoxon rank-sum is used to compute significance of the AUPR difference with 1 asterisk indicating pval < 0.05). (B) Difference in the performance of these two methods. Only the top 30 MF-GO terms on which *DeepFRI* > *DeepGOPlus* are shown.

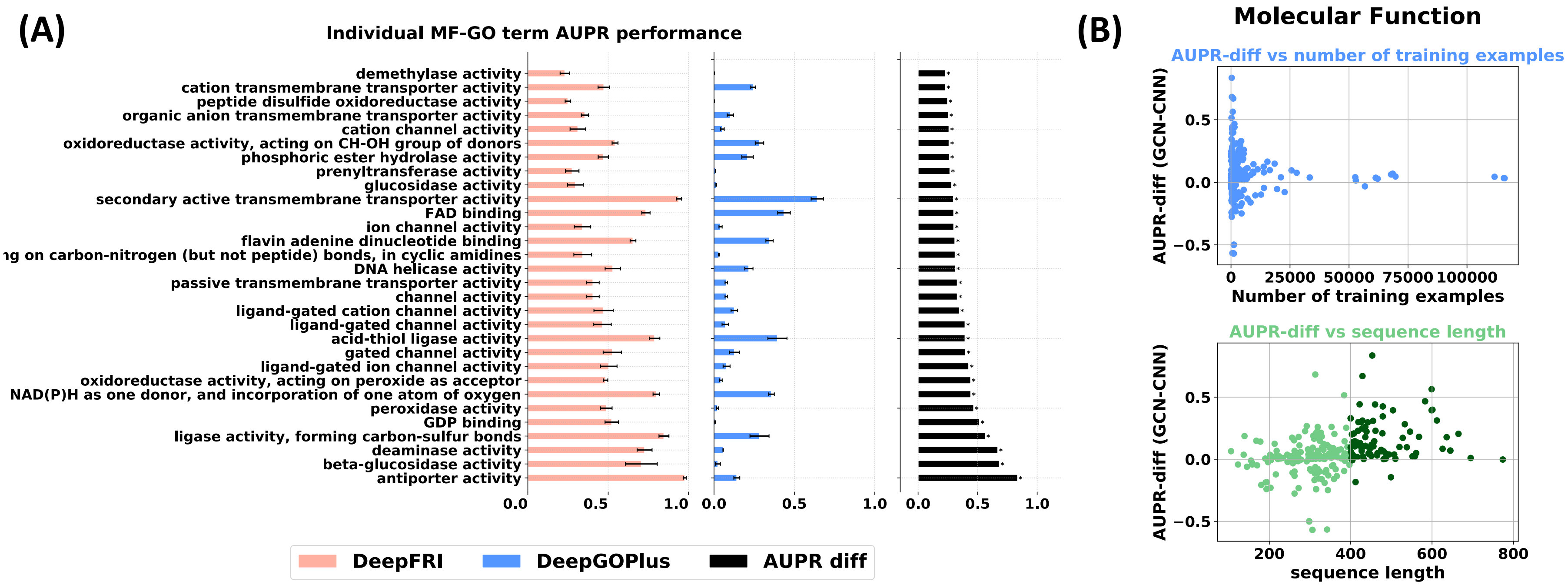

**Supplementary Figure 8.** Grad-CAM for "DNA binding" (GO:0003677) mapped onto the 3D structure of the test PDB chains annotated with "DNA binding". Their corresponding ROC curves measuring the overlap between the grad-CAM profile and DNA binding sites (retrieved from the BioLiP database) are shown in the bottom right corner.

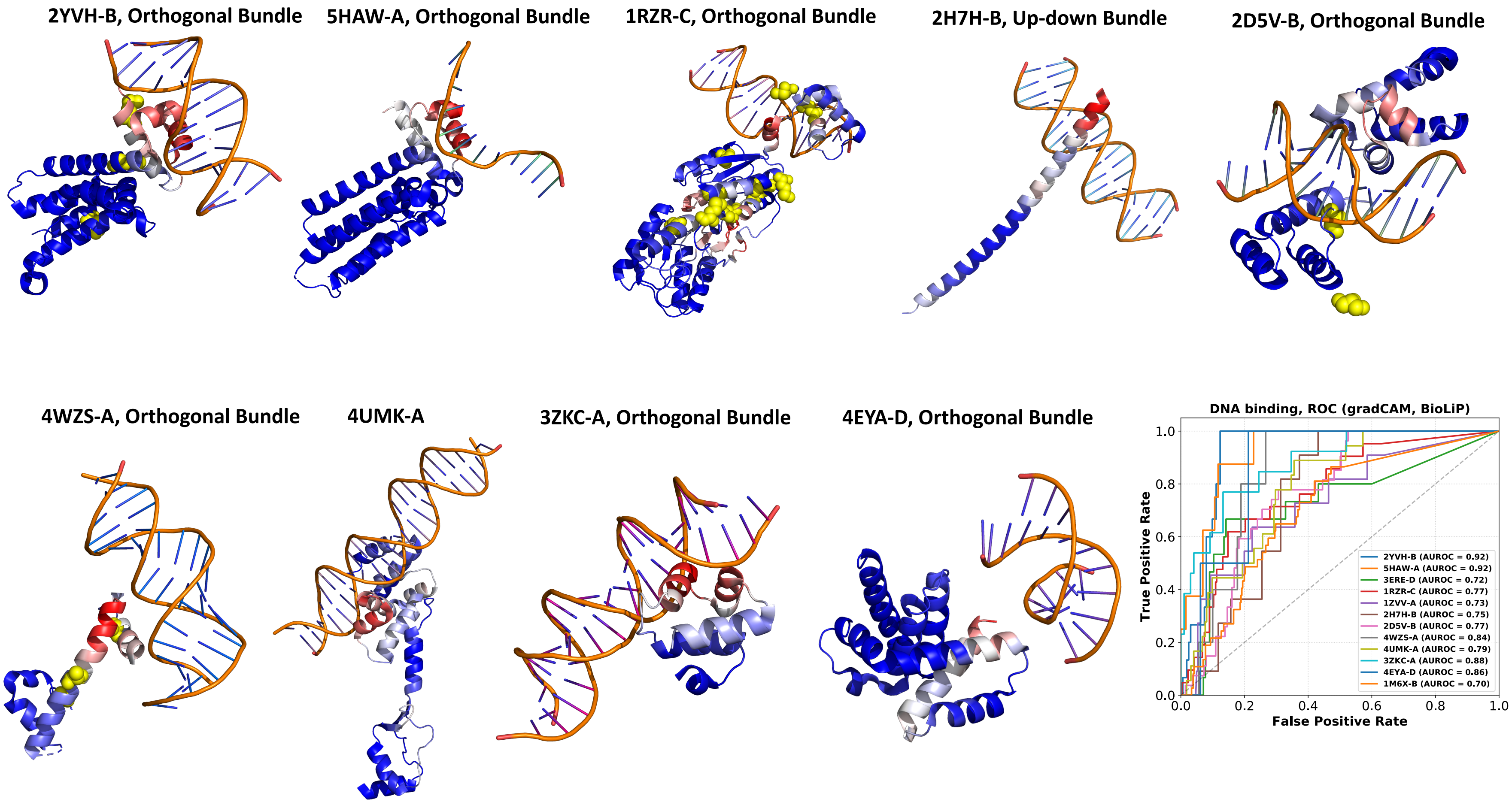

**Supplementary Figure 9.** Grad-CAM for "ATP binding" (GO:0005524) mapped onto the 3D structure of the test PDB chains annotated with "ATP binding". Their corresponding ROC curves measuring the overlap between the grad-CAM profile and ATP binding sites (retrieved from the BioLiP database) are shown in the bottom right corner.

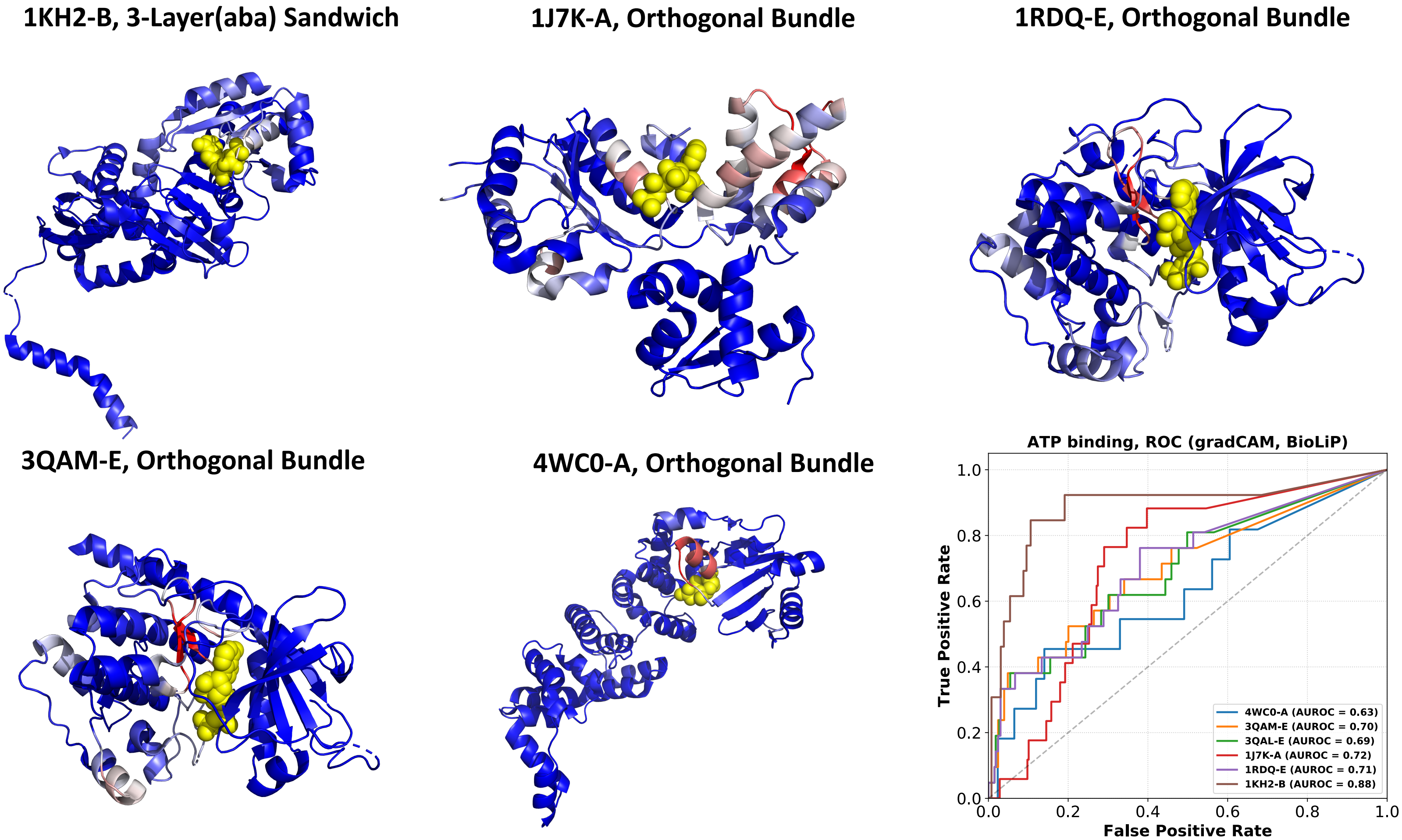

**Supplementary Figure 10.** Grad-CAM for "heme binding" (GO:0020037) mapped onto the 3D structure of the test PDB chains annotated with "heme binding". Their corresponding ROC curves measuring the overlap between the grad-CAM profile and HEM binding sites (retrieved from the BioLiP database) are shown in the bottom right corner.

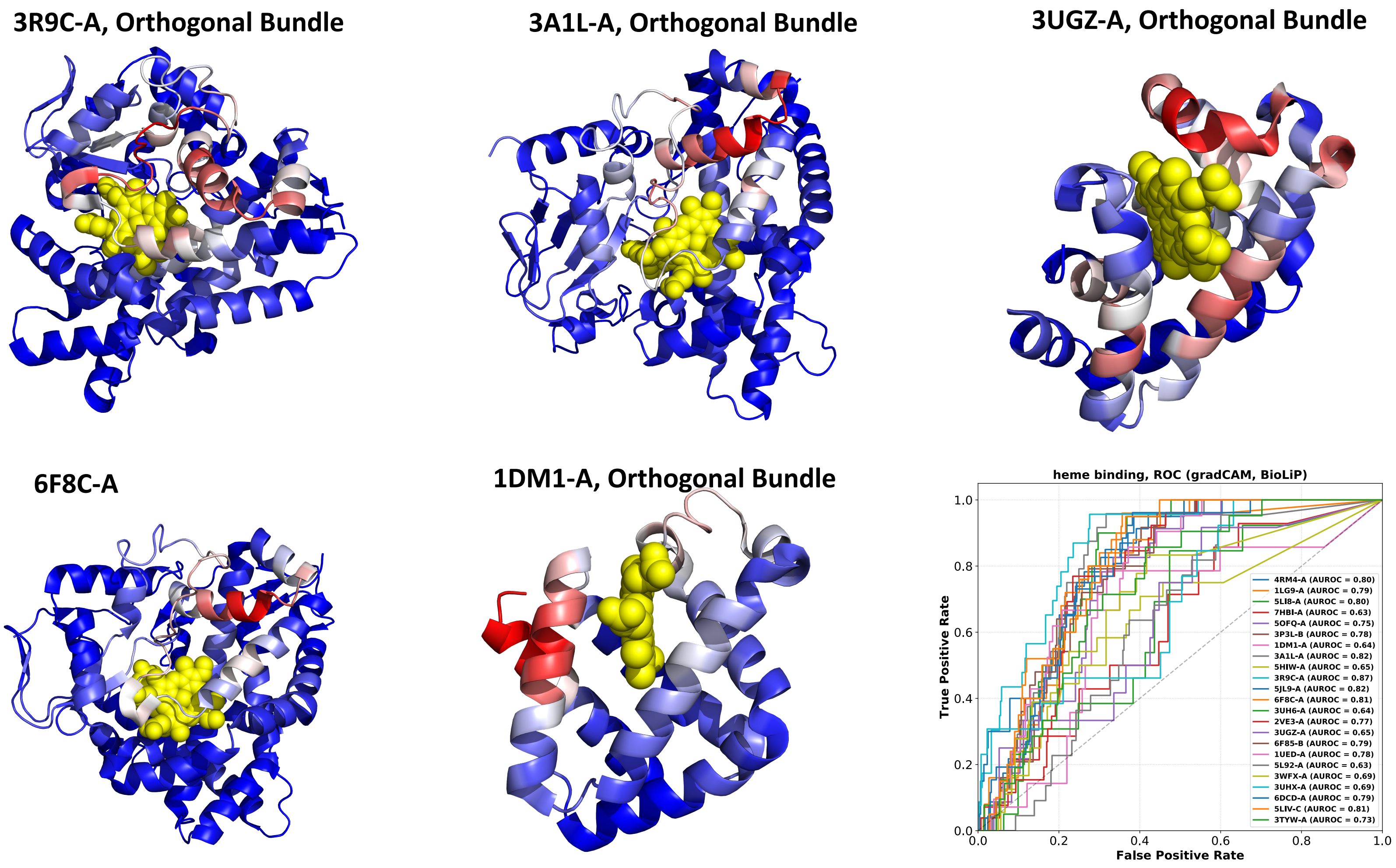

**Supplementary Figure 11.** Grad-CAM for "iron-sulfur cluster binding" (GO:0051536) mapped onto the 3D structure of the test PDB chains annotated with "iron-sulfur cluster binding". Their corresponding ROC curves measuring the overlap between the grad-CAM profile and SF4 binding sites (retrieved from the BioLiP database) are shown in the bottom right corner.

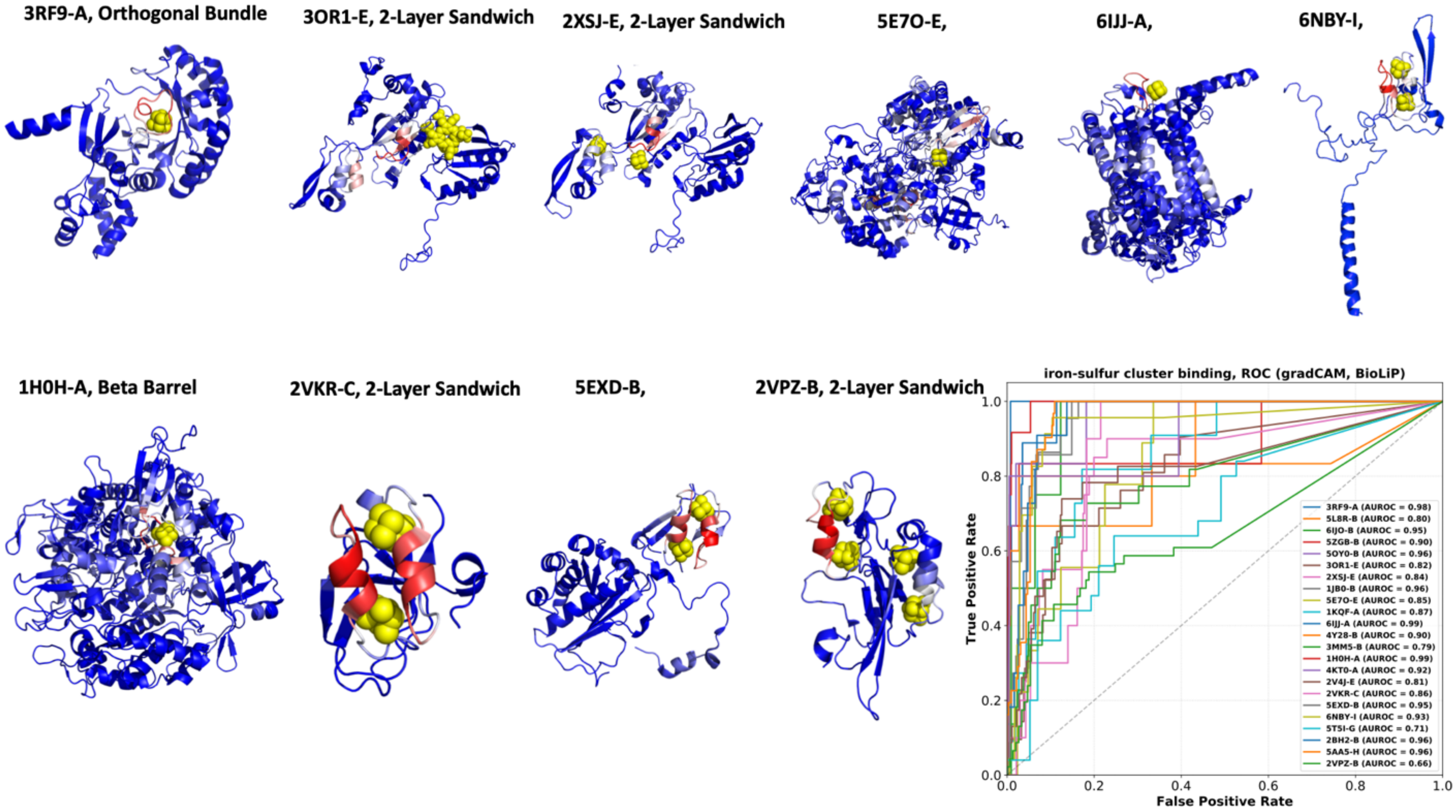

**Supplementary Figure 12.** Grad-CAM for "magnesium ion binding" (GO:0000287) mapped onto the 3D structure of the test PDB chains annotated with "magnesium ion binding". Their corresponding ROC curves measuring the overlap between the grad-CAM profile and MG binding sites (retrieved from the BioLiP database) are shown in the bottom right corner.

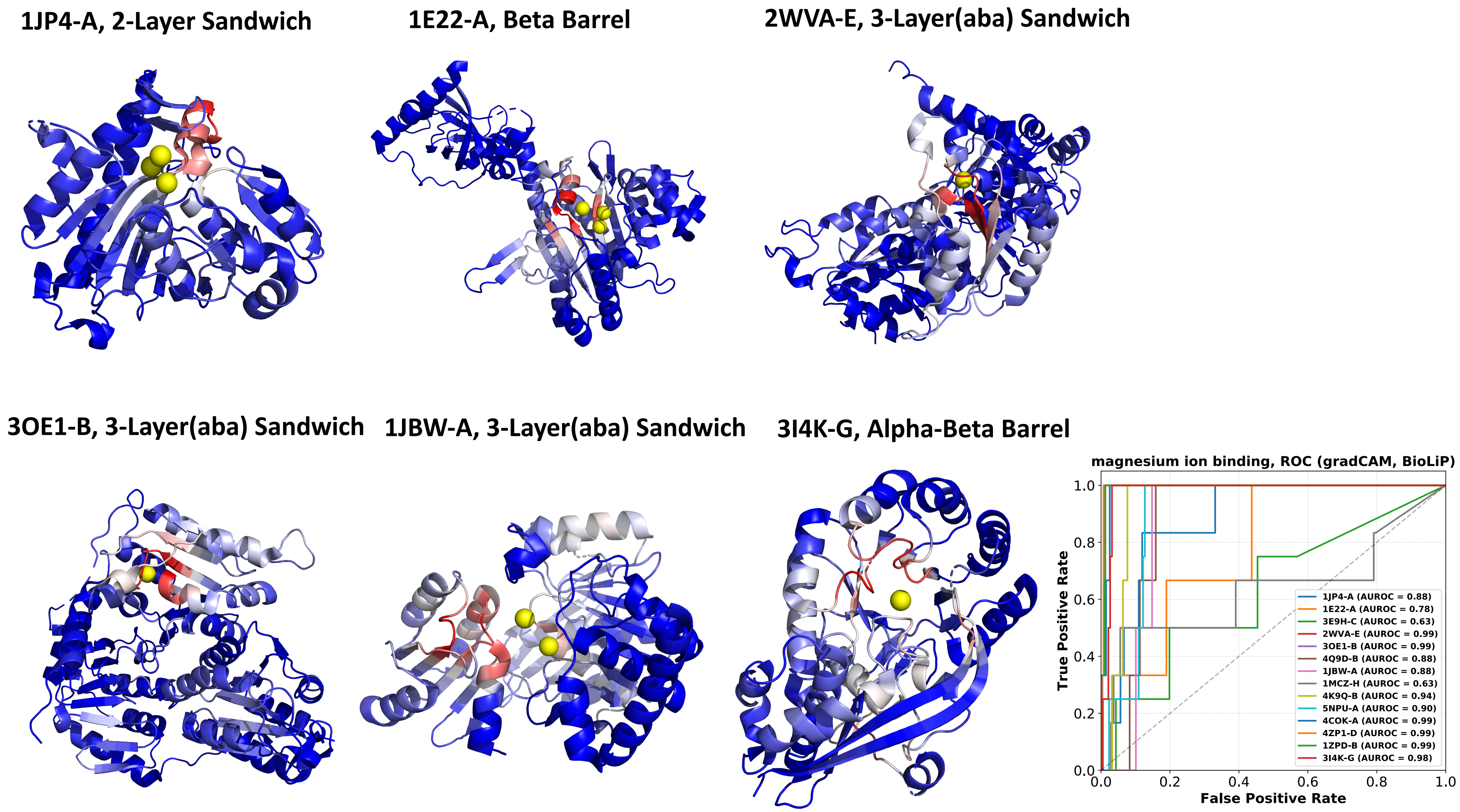

**Supplementary Figure 13.** Grad-CAM for "iron ion binding" (GO:0005506) mapped onto the 3D structure of the test PDB chains annotated with "iron ion binding". Their corresponding ROC curves measuring the overlap between the grad-CAM profile and FE binding sites (retrieved from the BioLiP database) are shown in the bottom right corner.

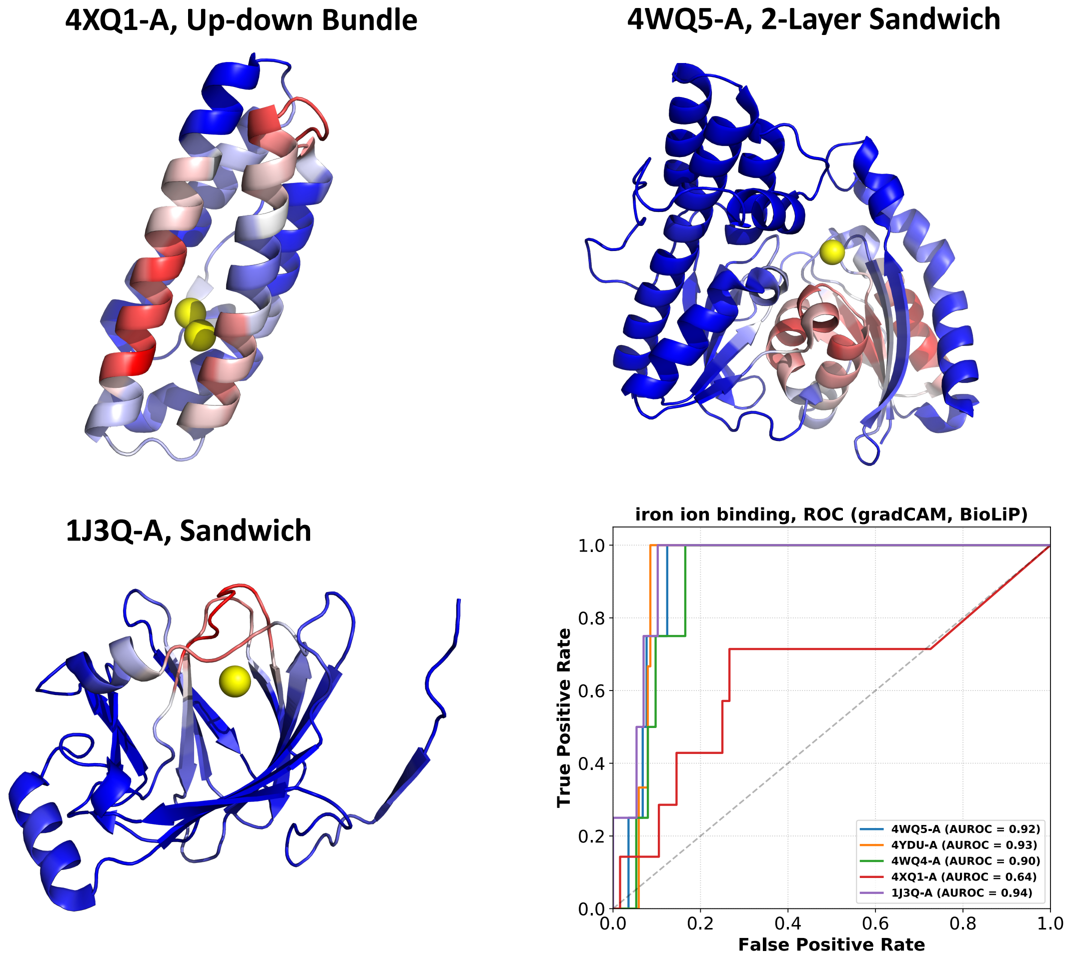

**Supplementary Figure 14.** Grad-CAM for "zinc ion binding" (GO:0008270) mapped onto the 3D structure of the test PDB chains annotated with "zinc ion binding". Their corresponding ROC curves measuring the overlap between the grad-CAM profile and ZN binding sites (retrieved from the BioLiP database) are shown in the bottom right corner.

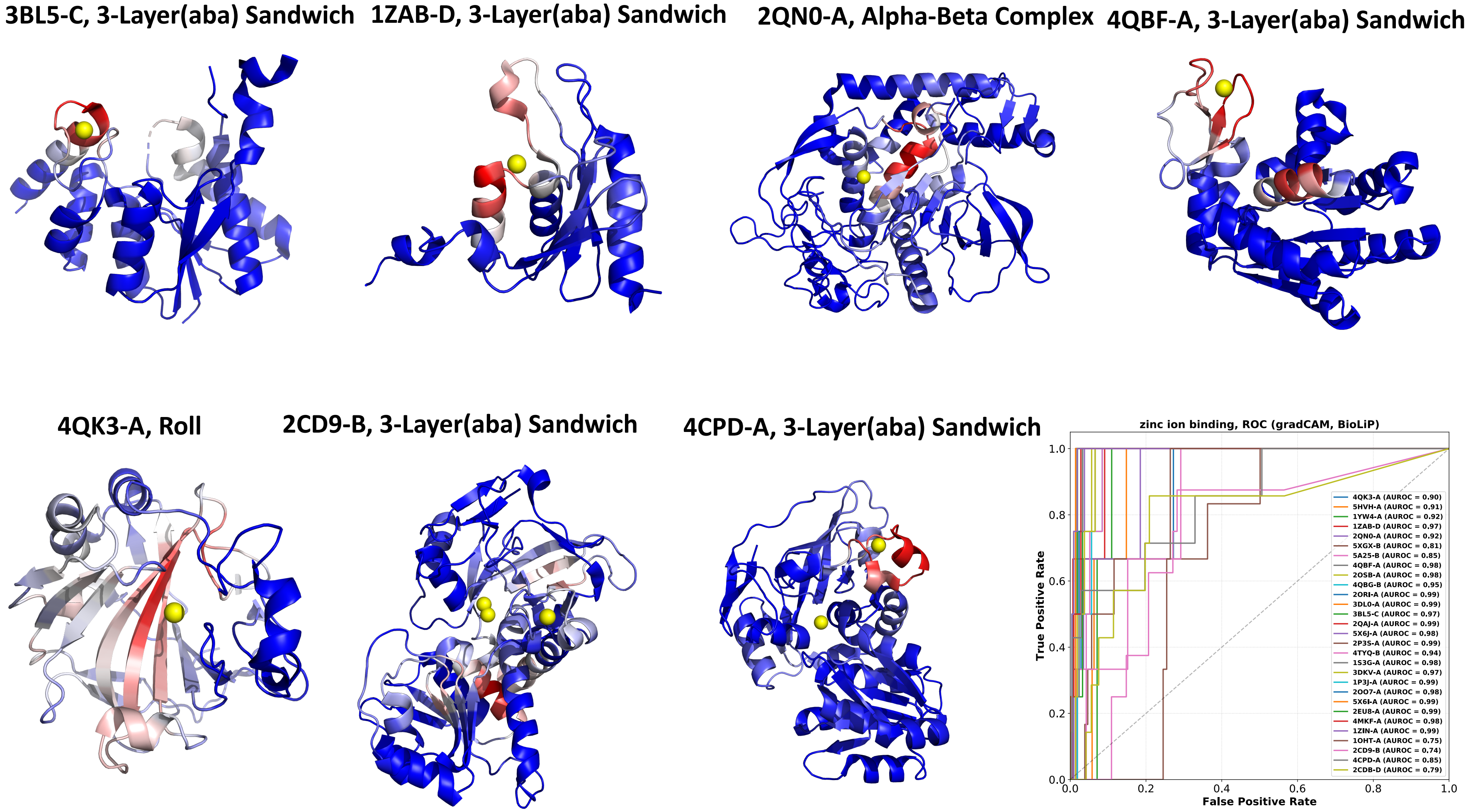

**Supplementary Figure 15**. Grad-CAM for “calcium ion binding” (GO:0005509) mapped onto the 3D structure of the test PDB chains annotated with “calcium ion binding”. Their corresponding ROC curves measuring the overlap between the grad-CAM profile and CA binding sites (retrieved from the BioLiP database) are shown in the bottom right corner.

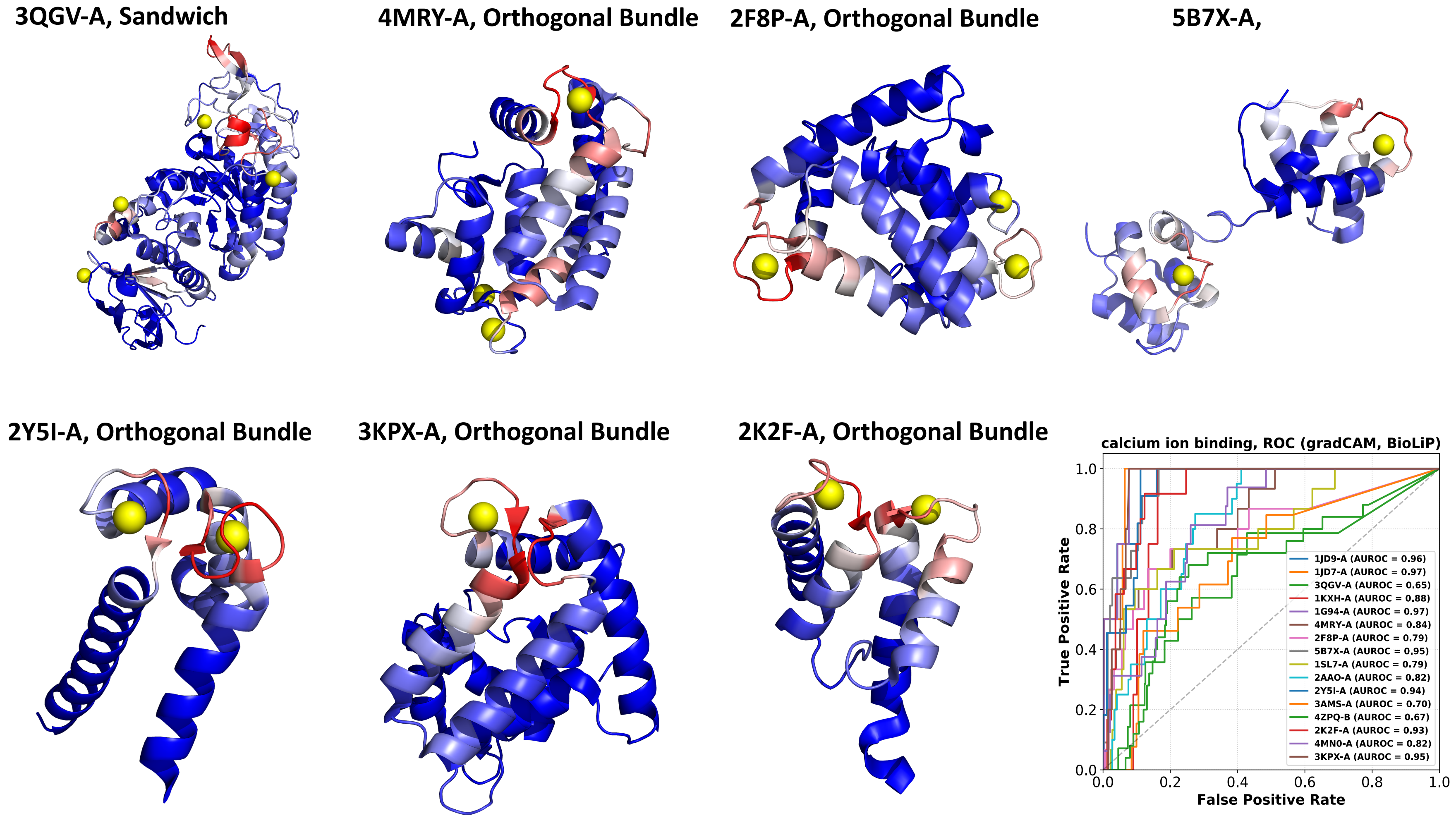

**Supplementary Figure 16.** Sensitivity vs. false-positive rate curve for DeepFRI in predicting catalytic residues. ROC measures the overlap between the grad-CAM profile and know catalytic sites (hand curated from CSA database) for 38 evolutionary divergent enzymes obtained from ResBoost paper.

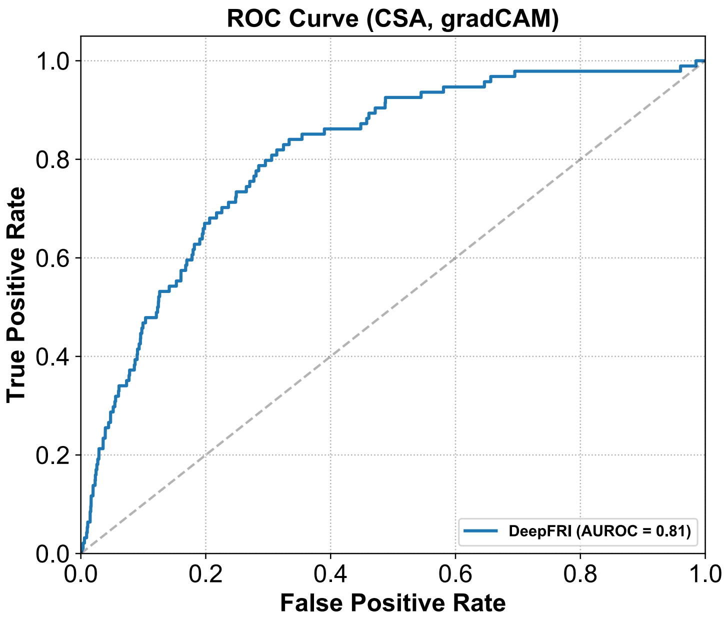

**Supplementary Figure 17**. Temporal holdout validation. (A) Average protein-centric F-max and term-centric AUPR values of our method in comparison to the CAFA-like *BLAST* baseline and *DeepGOPlus* applied on temporal holdout test PDB chains. The values are averaged over 100 bootstraps of the test set. Error bars represent standard deviation of the mean; asterisks indicate where the performance of *DeepFRI* is significantly better than the performance of *DeepGOPlus* (two-sided Wilcoxon rank-sum is used to compute significance with 3 asterisks indicating pval < 0.001); (B) PDB chains correctly annotated with our method (prediction score > 0.5) with very low *BLAST* and *DeepGOPlus* prediction scores; the low scores indicate the inability of *BLAST* and *DeepGOPlust* to correctly infer their GO terms. These PDB chains were selected because they have ligand-binding information in *BioLiP* that allows us to validate our Class Activation Mapping identification of functional sites on protein sequences and structures; (C) Grad-CAM profile mapped onto 3D stricture of the proteins in the Table.

**(A)**

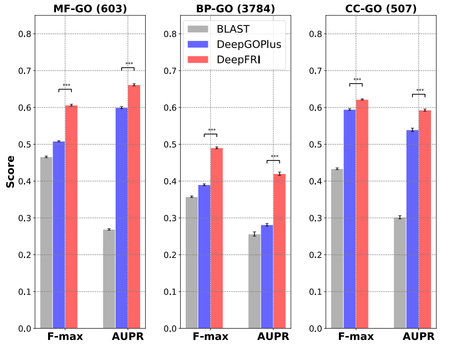


**(B)**

| PDB-chain | GO term | GO name | BLAST | DeepGO | DeepFRI |
|---|---|---|---|---|---|
| 6AGZ-B | GO:0010181 | FMN binding | 0.35 | 0.01 | 1.00 |
| 6Q72-E | GO:0070279 | vitamin B6 binding | 0.00 | 0.09 | 1.00 |
| 6E6B-A | GO:0005509 | calcium ion binding | 0.45 | 0.98 | 0.99 |
| 6OR8-A | GO:0051539 | 4 iron, 4 sulfur cluster binding | 0.00 | 0.35 | 0.98 |
| 6NHG-E | GO:0051537 | 2 iron, 2 sulfur cluster binding | 0.00 | 0.25 | 0.98 |
| 6RTD-A | GO:0020037 | heme binding | 0.00 | 0.21 | 0.87 |
| 6GTJ-A | GO:0005507 | copper ion binding | 0.26 | 0.10 | 0.87 |
| 6ONS-A | GO:0051539 | 4 iron, 4 sulfur cluster binding | 0.47 | 0.10 | 0.86 |
| 6IWP-A | GO:0008289 | lipid binding | 0.41 | 0.06 | 0.85 |
| 6JO6-A | GO:0051536 | iron-sulfur cluster binding | 0.00 | 0.01 | 0.79 |
| 6OHK-A | GO:0010181 | FMN binding | 0.37 | 0.02 | 0.79 |
| 6AIR-A | GO:0051536 | iron-sulfur cluster binding | 0.00 | 0.27 | 0.78 |
| 6S0O-D | GO:0005507 | copper ion binding | 0.34 | 0.04 | 0.73 |
| 6DE9-A | GO:0051536 | iron-sulfur cluster binding | 0.00 | 0.02 | 0.69 |
| 6NCO-A | GO:0008289 | lipid binding | 0.00 | 0.06 | 0.56 |
| 6S6X-J | GO:0051539 | 4 iron, 4 sulfur cluster binding | 0.00 | 0.06 | 0.52 |

**(C)**

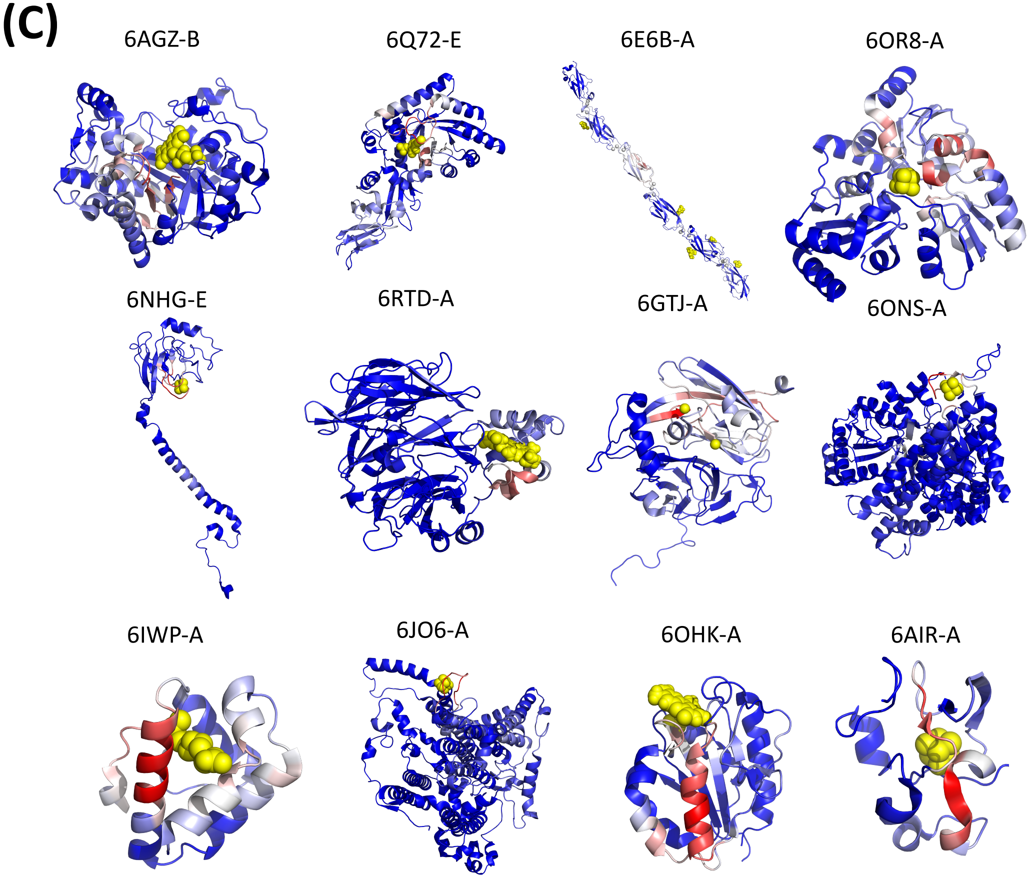

**Supplementary Figure 18**. (A) Performance of our method in comparison to state- of-the-art CNN (*DeepGO*) and *BLAST* baseline trained with different sequence features ("1HOT" - 26-dimensional binary one-hot encoding of residues, "LM" - features from the pretrained LSTM Language Model) and trained using protein chains with experimental (EXP) only and electronically inferred (EXP+IEA) MF-GO annotations. Again, all test proteins used to compose this metric were annotated with EXP evidence codes. (B) Performance of our model trained on CA-CA contact maps with experimental (EXP) annotations and evaluated on test set composed on CA-CA contact maps ("cmap"), generated contact maps with random contacts ("rand") and contact maps with not contacts except for self-loops ("diag"). The AUPR values are averaged over 100 bootstraps of the test set. Error bars represent standard deviation of the mean. Asterisks indicate where the performance of our method trained on CA-CA contact maps is significantly better than its' performance trained on "rand" and "diag". Two-sided Wilcoxon rank-sum is used to compute significance with 2 asterisks indicating pval < 0.01.

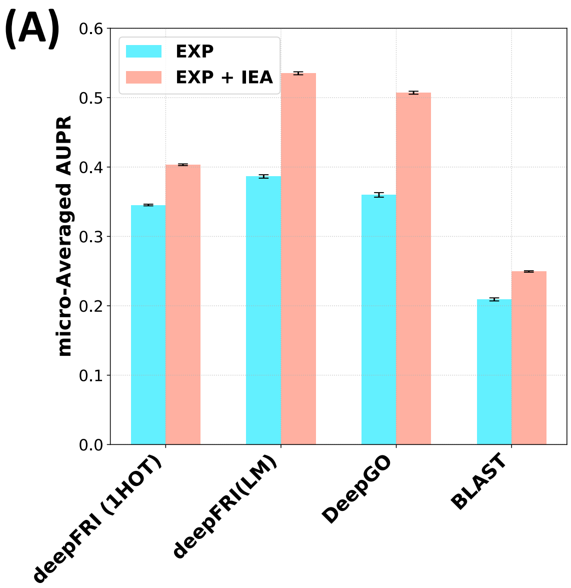


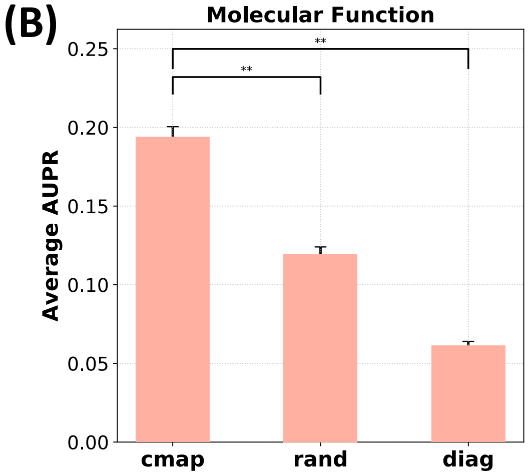

**Supplementary Figure 19**. (Top) Distribution of PDB chains across different protein families (Pfam) in the training (left) and the test set (right). (Bottom) Protein-centric Precision-Recall curves showing the performance of DeepFRI in comparison to FunFams and DeepGO averaged over test PDB chains belonging to the top 6 protein families in the test set.

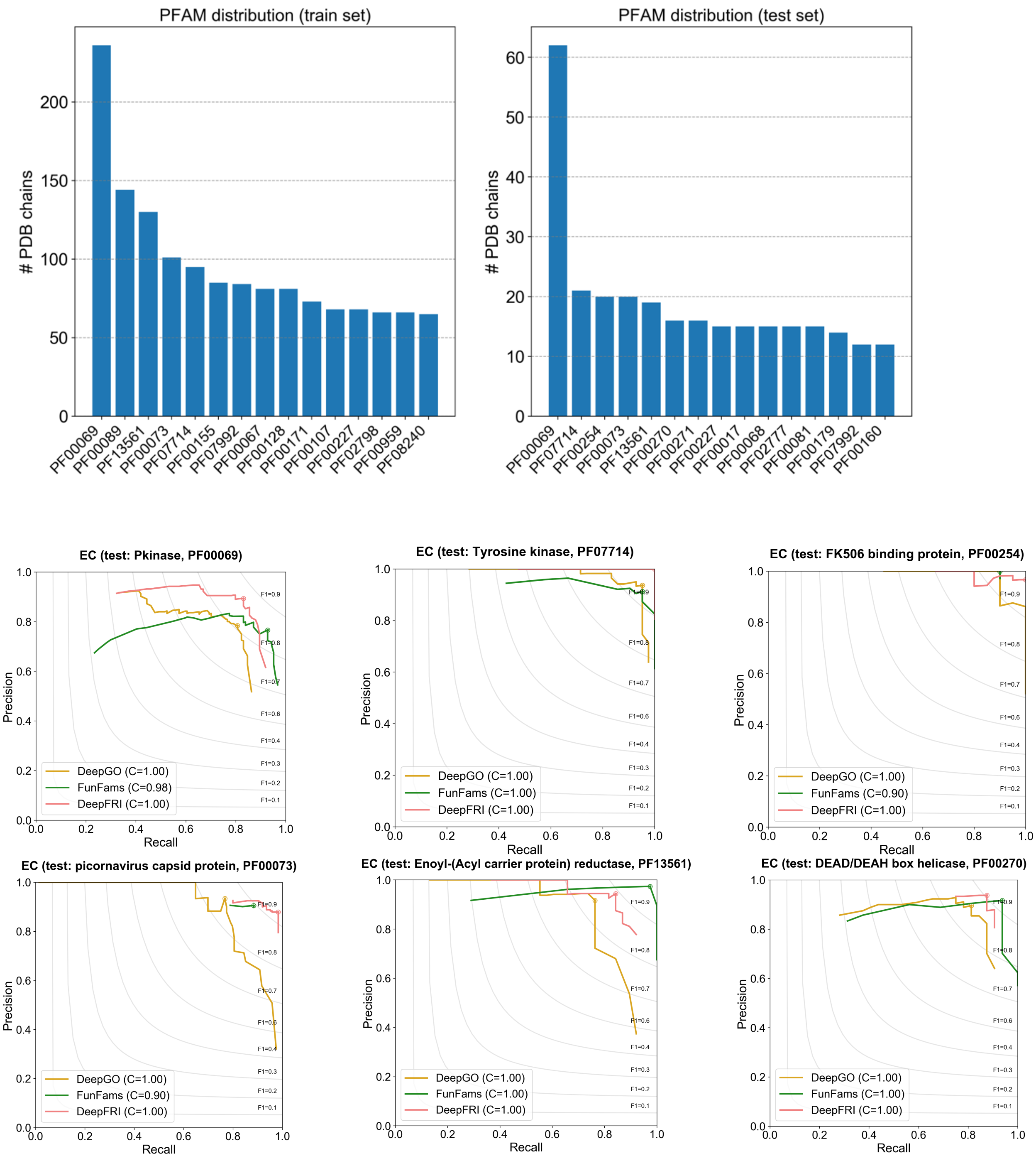

**Supplementary Figure 20**. Performance of our method on a test set of proteins from different organisms measured as protein-centric $F_{max}$ score summarized under 10 bootstraps of the test set. Error bars represent standard deviation of the mean. See Supplementary Table 2 for the number of test proteins in each set.

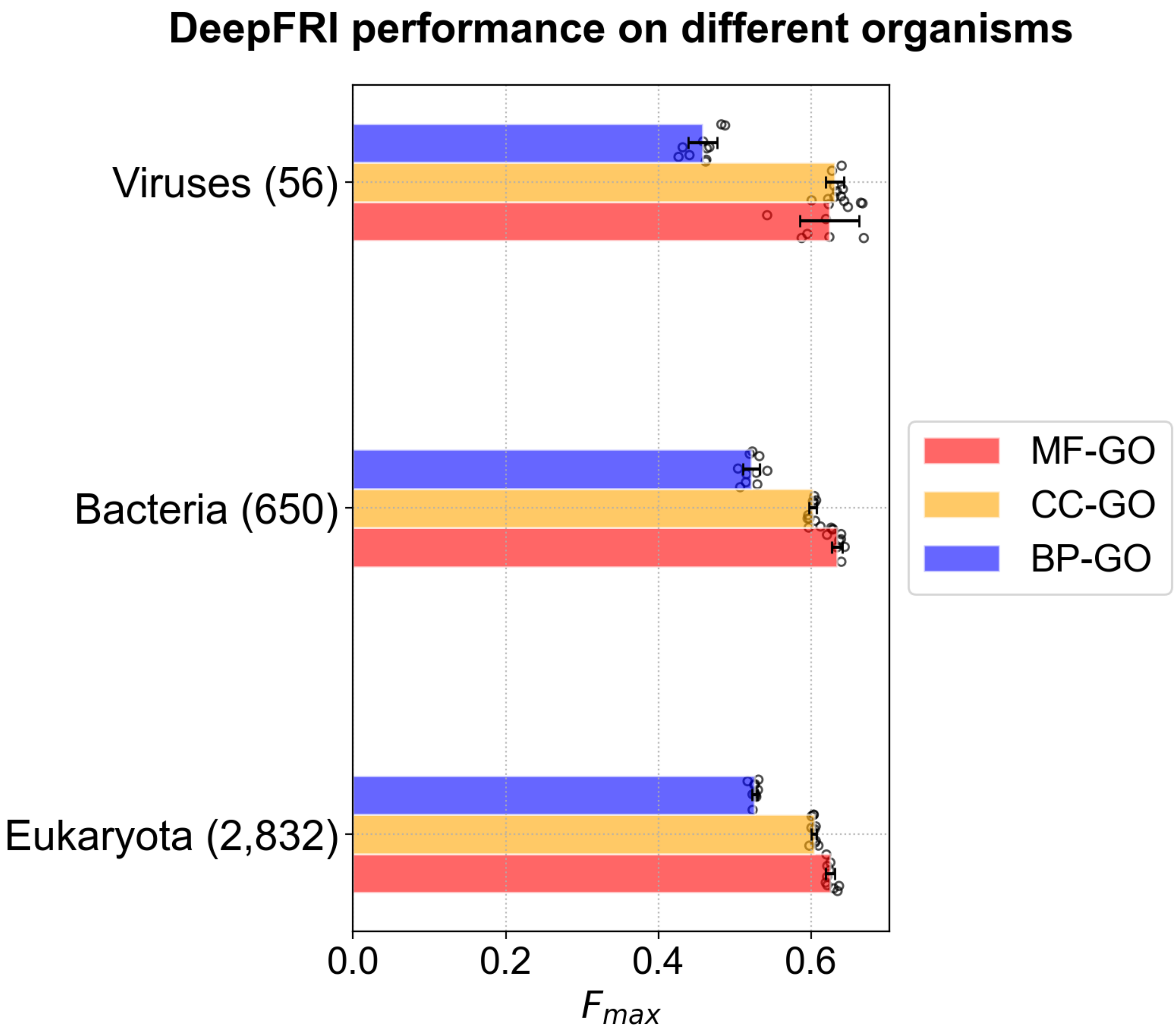

**Supplementary Figure 21**. Protein-centric Precision-Recall curves showing the performance of DeepFRI in comparison to DeepGO and BLAST baseline averaged over PDB chains belonging to the top 23 largest protein families (PF00089, PF07654, PF07686, PF00072, PF00128, PF13499, PF00042, PF07714, PF13561, PF00171, PF00155, PF00073, PF00005, PF00069, PF00067, PF00004, PF00076, PF00085, PF07992, PF00071, PF01547, PF00440, PF00400) in the test set.

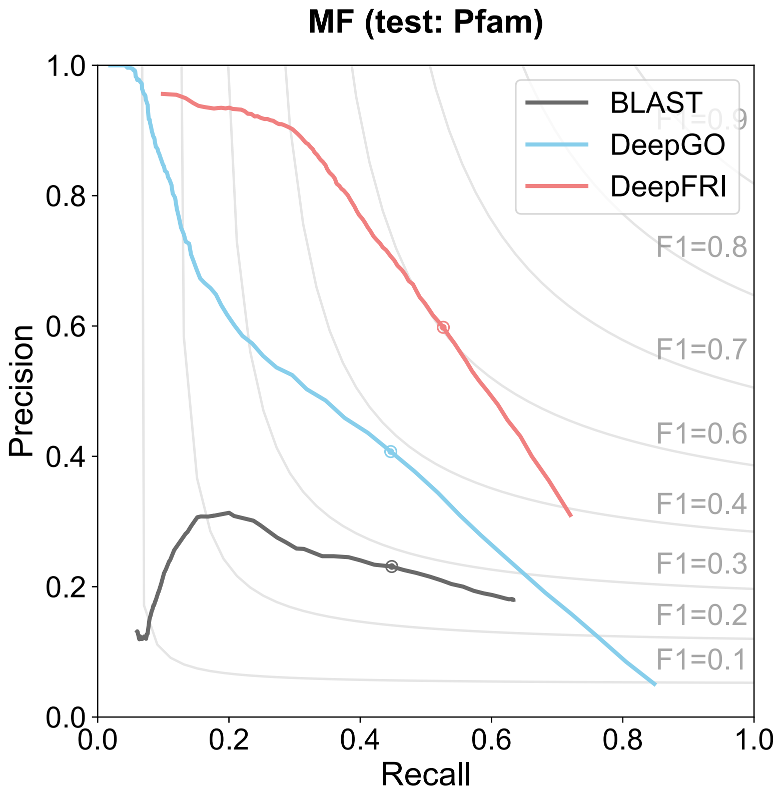

**Supplementary Figure 22**. Protein-centric Precision-Recall curves showing the performance of DeepFRI in comparison to DeepGO and BLAST baseline averaged over PDB chains belonging to the top 4 largest CATH folds: 3.20.20 (TIM barrel), 2.60.40 (Immunoglobulin-like), 2.60.120 (Jelly Rolls), 3.30.70 (Alpha-Beta Plaits) in the test set.

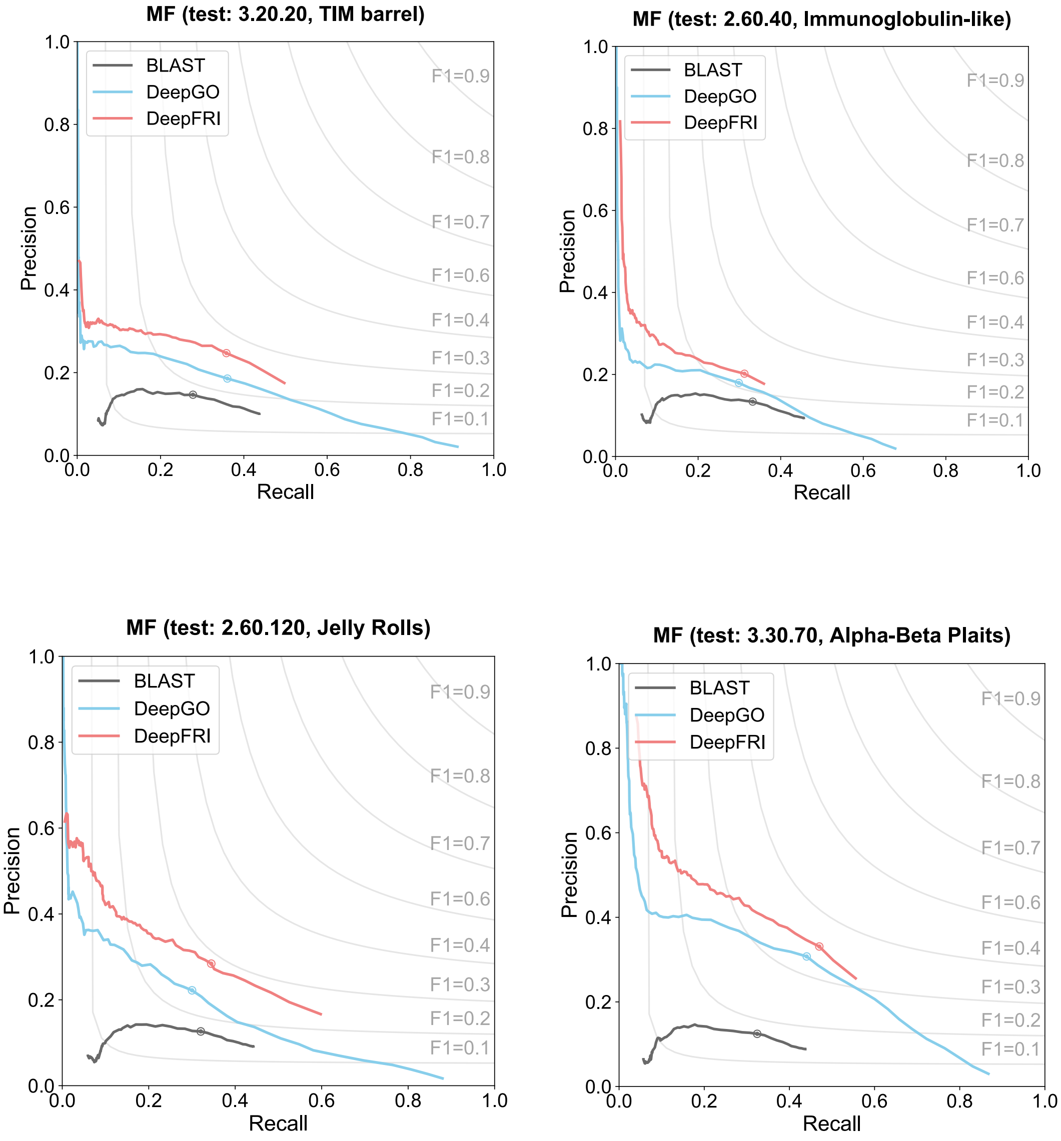

**Supplementary Table 1**. Table showing the distribution of PDB chains in the test set with 30%, 40%, 50%, 70% and 95% sequence identity to the training set (columns 2-6) and in different organisms (columns 7-9).

| Data | Annotations | Train | Test | Validation | # terms |
|---|---|---|---|---|---|
| PDB | MF | 29,902 | 3,416 | 3,323 | 489 |
| | BP | 29,902 | 3,416 | 3,323 | 1,943 |
| | CC | 29,902 | 3,416 | 3,323 | 320 |
| | EC | 15,551 | 1,919 | 1,729 | 538 |
| SWISS-MODEL | MF | 220,297 | 3,416 | 24,478 | 489 |
| | BP | 220,297 | 3,416 | 24,478 | 1,943 |
| | CC | 220,297 | 3,416 | 24,478 | 320 |
| | EC | 122,697 | 1,919 | 13,633 | 538 |

**Supplementary Table 2**. Table showing the number of PDB & SWISS-MODEL chains in training, test and validation sets in GO and EC classification systems.

| Test | 30% | 40% | 50% | 70% | 95% | Eukaryote | Bacteria | Viruses |
|---|---|---|---|---|---|---|---|---|
| GO | 1,717 | 1,937 | 2,199 | 2,733 | 3,416 | 2,832 | 650 | 56 |
| EC | 720 | 902 | 1,117 | 1,476 | 1,919 | 944 | 787 | 166 |
