# Supplementary material for: Structure-based protein function prediction using graph convolutional networks
Source: Nat Commun. 2021 May 26;12:3168. doi: 10.1038/s41467-021-23303-9 (PMC8155034; doi:10.1038/s41467-021-23303-9)
Supplement: Supplementary file 2 — Description of Additional Supplementary Files [file 41467_2021_23303_MOESM2_ESM.pdf]

Description of Additional Supplementary Files

Supplementary Data 1: List of unannotated PDB chains with our predicted functions.

Supplementary Data 2: List of unannotated SWISS-MODEL chains with our predicted functions.
